# Meta-Omics Reveals Genetic Flexibility of Diatom Nitrogen Transporters in Response to Environmental Changes

**DOI:** 10.1093/molbev/msz157

**Published:** 2019-07-01

**Authors:** Greta Busseni, Fabio Rocha Jimenez Vieira, Alberto Amato, Eric Pelletier, Juan J Pierella Karlusich, Maria I Ferrante, Patrick Wincker, Alessandra Rogato, Chris Bowler, Remo Sanges, Luigi Maiorano, Maurizio Chiurazzi, Maurizio Ribera d’Alcalà, Luigi Caputi, Daniele Iudicone

**Affiliations:** 1 Stazione Zoologica Anton Dohrn, Naples, Italy; 2 Institut de Biologie de l’Ecole Normale Supérieure (IBENS), Ecole Normale Supérieure, CNRS, INSERM, PSL Université Paris, Paris, France; 3 Laboratoire de Physiologie Cellulaire et Végétale, Univ. Grenoble Alpes, CEA, INRA, CNRS. BIG, Grenoble Cedex 9, France; 4 Génomique Métabolique, Genoscope, Institut François Jacob, CEA, CNRS, Univ Evry, Université Paris-Saclay, Evry, France; 5 FR2022/Tara Oceans-GOSEE, Paris, France; 6 Institute of Biosciences and BioResources, CNR, Naples, Italy; 7 Scuola Internazionale Superiore di Studi Avanzati (SISSA), Trieste, Italy; 8 Dipartimento di Biologia e Biotecnologie “Charles Darwin”, Università di Roma “La Sapienza”, Roma, Italy

**Keywords:** diatoms, meta-omics, nitrogen transporters, machine learning

## Abstract

Diatoms (Bacillariophyta), one of the most abundant and diverse groups of marine phytoplankton, respond rapidly to the supply of new nutrients, often out-competing other phytoplankton. Herein, we integrated analyses of the evolution, distribution, and expression modulation of two gene families involved in diatom nitrogen uptake (Di*AMT1* and Di*NRT2*), in order to infer the main drivers of divergence in a key functional trait of phytoplankton. Our results suggest that major steps in the evolution of the two gene families reflected key events triggering diatom radiation and diversification. Their expression is modulated in the contemporary ocean by seawater temperature, nitrate, and iron concentrations. Moreover, the differences in diversity and expression of these gene families throughout the water column hint at a possible link with bacterial activity. This study represents a proof-of-concept of how a holistic approach may shed light on the functional biology of organisms in their natural environment.

## Introduction

Uptake, translocation, and storage/redistribution of inorganic nitrogen (N) are essential processes for all photosynthetic organisms. These activities rely principally on three protein families, the nitrate/peptide (NPF), nitrite–nitrate (NRT), and the ammonium (AMT) transporters belonging to the Major Facilitator Superfamily (MFS) (Pao et al. 1998).

While in terrestrial multicellular phototrophs the localization and regulation of these transporter proteins have been widely studied (e.g., Wang et al. 2018), for marine unicellular phototrophs, whose environment is characterized by low and fluctuating concentrations of their substrates, exploration is still in its infancy. Physiological studies have yielded exhaustive reviews of uptake kinetics in different environments but only recently have molecular mechanisms been addressed (e.g., Rogato et al. 2015; McCarthy et al. 2017 for diatoms). Moreover, our knowledge so far derives mostly from laboratory experiments on model organisms, and only scant information is available on the regulation of N-transporters in the natural environment.

This study focuses on diatoms (Bacillariophyta), one of the most abundant and diverse groups of marine phytoplankton. One peculiarity of this group is to rapidly respond to the supply of new nutrients (Caputi et al. 2019) and to typically out-compete other phytoplankton when nutrient limiting conditions are removed. Diatoms possess a complex, yet understudied, repertoire of proteins involved in N uptake and internal management (Glibert et al. 2016). The transcriptional response of diatoms to N deprivation generally consists in regulating genes involved in N uptake and assimilation, as well as using alternative N sources (Alipanah et al. 2015; McCarthy et al. 2017). In particular, nitrate (NO_3_^−^) and ammonium (${\text{NH}}_{4}^{\text{+}}$) transporter proteins should be able to cope with, and compensate for, the fluctuations in concentration usually observed in the ocean (Rogato et al. 2015 and references therein). These depend on vertical exchanges driven by physical processes and on phytoplankton and microbial activities (see Capone et al. 2008), which produce large geographical variations in N fluxes to the photic zone. Vertical transport of NO_3_^−^ has a major role in determining regional differences in N availability, since most of the regenerated NO_3_^−^ and NO_2_^−^ produced by nitrification reaches the photic zone via vertical transport (Capone et al. 2008). However, because their concentrations are always in the micromolar range, diatom transporters are most likely to rely on the high-affinity transporter classes. In fact, biphasic saturable and nonsaturable kinetics of uptake have been reported for algal cultures only at concentrations ∼300 μM NO_3_^−^, which may be found in eutrophic coastal systems but that far exceed normal oceanic concentrations, which reach a maximum of ∼45 μM (Glibert et al. 2016). One NO_3_^−^ and two ${\text{NH}}_{4}^{\text{+}}$ high affinity transporter gene families are known, *NRT2* and *AMT1/AMT2*, respectively. These appear to be monophyletic in land plants (von Wittgenstein et al. 2014) but diatoms have been recently reported to lack AMT2 genes (Rogato et al. 2015). Diatoms typically contain three to six *NRT2* and five to seven *AMT1* transporters per genome (Rogato et al. 2015). Most *NRT2* genes are up-regulated in conditions of N starvation while *AMT1* expression shows less clear modulation patterns in the same conditions (Rogato et al. 2015; Glibert et al. 2016; McCarthy et al. 2017). Other internal and external cues may play a role in the regulation of NO_3_^−^ and ${\text{NH}}_{4}^{\text{+}}$ uptake. For example, light (Bender et al. 2014), temperature (Lomas and Glibert 1999), turbulence (dell’Aquila et al. 2017) and grazing activity (Amato et al. 2018) were found to modulate expression of N uptake genes. At the same time, internal processes such as the cell cycle (Hildebrand and Dahlin 2000) and the internal nutritional status of the cell (Allen et al. 2011) have also been suggested to have a role in this modulation.

Considering all the above, N uptake might have had an important role in allowing diatom success in the paleo and contemporary ocean. If so, to what extent and how the variable number of Di*AMT1* and Di*NRT2* genes contributes to that role is not known. Likewise, whether and how this variety reflects a differential usage of the genes is mostly unknown. As diatoms are present in all oceanic regions and at different depths, we assumed, as a starting hypothesis, that the repertoire and the expression of different N transporter genes would mirror horizontal and vertical environmental differences. Once identified, and considering the importance of N transport for diatom survival, it should be possible to predict how the diversity of this trait would determine the diatom response in a changing ocean. To address these issues, we used the *Tara* Oceans data set (De Vargas et al. 2015; Carradec et al. 2018), which provides a unique combination of environmental, metagenome, and metatranscriptome data.

## Results

### The Evolution of Diatom N Transporters Is Characterized by a Complex Pattern of Differential Losses and Duplications

We performed a preliminary search for putative Di*AMT2* in both the *Tara* Oceans eukaryote unigene catalog (MATOU—Carradec et al. 2018) and in the Marine Microbial Eukaryote Transcriptome Sequencing Project (MMETSP—Keeling et al. 2014). Results confirm the previously reported absence of *AMT2* genes in diatoms (Rogato et al. 2015).

**Fig. 1.**
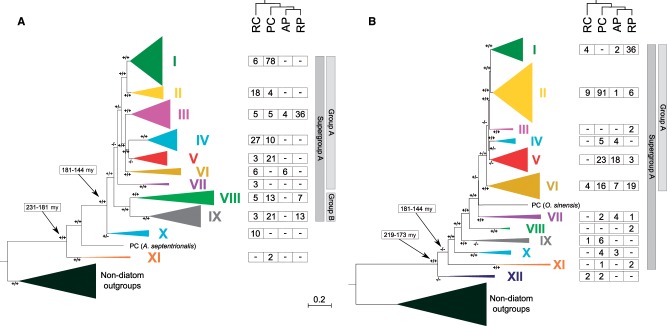
Phylogenetic relationships of diatom AMT1 (*A*) and NRT2 (*B*) protein sequences. The first “+” positioned close to a branch indicates a Shimodaira–Hasegawa (SH) support >50% for that clade as calculated by aML, while the second “+” indicates a posterior probability >0.5 as calculated by BI. A number of 11 phylogenetic clades were identified in DiAMT1 phylogenetic tree, while 12 clades were found for DiNRT2. All the branches within clades have been collapsed for simplicity and the number of sequences corresponding to each major diatom group is indicated (RC, radial-centric-basal-Coscinodiscophyceae; and the three Bacillariophytina groups: PC, polar-centric-Mediophyceae; AP, araphid-pennate; RP, raphid-pennate). Black arrows indicate main groups’ divergence dates in millions of years (My—Medlin 2016). The trees were rooted using nondiatom sequences as outgroups (supplementary data files S5 and S6, Supplementary Material online), which have been collapsed as outgroup. Combining N transporter phylogenetic tree and the simplified phylogenies of diatoms (upper trees), we observe an evolutionary scenario characterized by several gene loss and duplication events (clades were color coded for later use).

To investigate the isoform diversity and taxonomic distribution of diatom N transporters in the global ocean, we searched for Di*AMT1* and Di*NRT2* sequences in the MATOU catalog (Carradec et al. 2018; supplementary data files S1 and S2, Supplementary Material online) using machine learning approaches and optimization techniques as implemented in DAMA/CLADE tools (see Materials and Methods; Bernardes et al.^2016^). 307 Di*AMT1* unigenes were identified from all diatom classes (28% from radial-centric-basal-Coscinodiscophyceae, 50% from polar-centric-Mediophyceae, 3% from araphid pennates, and 18% from raphid pennates) (fig. 1*A* and supplementary data files S3 and S4, Supplementary Material online). In a similar fashion, 281 Di*NRT2* unigenes were retrieved (6% from radial-centric-basal-Coscinodiscophyceae, 55% from polar-centric-Mediophyceae, 14% from araphid pennates, and 25% from raphid pennates) (fig. 1*B*). The inferred phylogenetic trees for the corresponding protein sequences are shown in figure 1.

The tree topology for *AMT1* indicates that Di*AMT1* sequences are monophyletic with respect to the nondiatom sequences and allow us to define clades I to XI (fig. 1*A* and supplementary data files S5 and S6, Supplementary Material online). Phylogenetic analyses suggest that ancestral diatoms, dated prior to the Coscinodiscophytina and Bacillariophytina radiation, likely already contained a rich repertoire of Di*AMT1*. The radial-centric-basal-Coscinodiscophyceae diatoms retained most of the Di*AMT1* subgroups; indeed, this group is represented in all the Supergroup A clades. On the other hand, the Bacillariophytina lineage most probably experienced, by gene loss, a gradual reduction of the Di*AMT1* repertoire.

The diatom portion of the *NRT2* tree is monophyletic, with no sign of lateral gene transfer (fig. 1*B*). Herein, we name Supergroup A the portion of the tree which includes clades I–XI, and Group A the portion of Supergroup A including clades I–VI (fig. 1*B*). Clade XII, containing Di*NRT2* sequences assigned to radial-centric-basal-Coscinodiscophyceae and to polar-centric-Mediophyceae diatoms, is basal to Supergroup A. The most parsimonious evolutionary scenario emerging from the tree postulates that five classes of Di*NRT2* were present in diatoms prior to the Coscinodiscophytina and Bacillariophytina radiation. In radial-centric-basal-Coscinodiscophyceae, this number was retained up to the present age, while in the Bacillariophytina lineage, one or more events of gene duplications occurred in the class Mediophyceae (represented in 10 out of 12 clades), possibly followed by specific gene loss events in araphid (represented in 7 out 12 clades) and raphid (represented in 8 out 12 clades) pennates.

The evolutionary history emerging from the tree topology indicates that Di*AMT1* and Di*NRT2* are characterized, similarly to other phytoplankton groups and in land plants (McDonald et al. 2010; von Wittgenstein et al. 2014), by differential losses and duplications, without any lateral gene transfer.

### Diatom AMT1 and NRT2 Transporters Are Structurally Conserved

To determine the structural features of diatom AMT1 and NRT2, we aligned the translated sequences retrieved from the *Tara* Oceans eukaryotic gene catalog and from sequenced genomes (Rogato et al. 2015; supplementary data files S7 and S8, Supplementary Material online) with 45 Di*AMT1* and 51 Di*NRT2* new sequences that we identified in the transcriptomes of 92 diatom species from the MMETSP (see Materials and Methods; Keeling et al. 2014).

DiAMT1 proteins are predicted to display the canonical 11 transmembrane (TM) domains with a N-out, C-in topology (Levitan et al. 2015), and possess 13 out of the 14 conserved amino acid residues reported to be functionally significant for ${\text{NH}}_{4}^{\text{+}}$ eukaryotic transport (Andrade and Einsle 2007). The LGTF signature of DiAMT1 (supplementary fig. S1*A*, Supplementary Material online) lies within the sixth TM domain, whereas the DiNRT2 GVELT signature (supplementary fig. S1*B*, Supplementary Material online) is inside the seventh TM domain. The presence of 12 TM domains has already been predicted in most of the DiNRT2 proteins (Rogato et al. 2015). These analyses suggest that, despite the specificity and variability of global oceans conditions, the mechanisms of transport of ${\text{NH}}_{4}^{\text{+}}$ and NO_3_^−^ are rather conserved in DiAMT1 and DiNRT2 proteins.

A functional diversification among the members of transporter families might be also associated to their subcellular localization. Accumulation of intracellular NO_3_^−^ (ICNO_3_) has been reported in both pelagic and benthic diatoms (Stief et al. 2013). This striking capacity may allow vacuolar NO_3_^−^ accumulation at two to three orders of magnitude higher than ambient concentrations. We performed subcellular targeting prediction analysis on a bulk of 109 DiNRT2 sequences >500 amino acids long, by exploiting the LocTree3 software that predicts the localization also via homology-based inference between proteins of known localization (supplementary data file S9, Supplementary Material online; Goldberg et al. 2014). The number of predicted diatom vacuolar-DiNRT2 sequences (25%) is about twice those predicted on a bulk of similar size (107 nondiatom sequences, 13%) including the sequences from 19 plant families NRT2 (25% vs. 13%; *P* = 0.036). Although bioinformatics predictions require experimental validation, these preliminary results suggest that high affinity transporters are crucial actors for vacuolar NO_3_^−^ loading and accumulation in diatoms.

A divergent sublocalization was not displayed by the DiAMT1 sequences, that were all predicted to be plasma membrane-located, a result consistent with the lack of reported storage mechanisms for ${\text{NH}}_{4}^{\text{+}}$ in the vacuole in diatoms (Glibert et al. 2016; McCarthy et al. 2017).

### Global Richness of Di*AMT1* and Di*NRT2* Clades Show Hotspots of Functional Diversity

The analysis of the *Tara* Oceans 18S ribosomal DNA database (De Vargas et al. 2015) highlighted strong taxonomical differences for diatoms across the ocean basins (Malviya et al. 2016). To assess a degree of diversity more related to functions, we measured clade-based richness on both Di*AMT1* and Di*NRT2* mRNA levels across the global ocean (supplementary fig. S1*C* and *D*, Supplementary Material online). Clade diversity in different sites ranges from one to ten clades present for both Di*AMT1* and Di*NRT2*, with the Mediterranean basin and the Indian Ocean (IO) characterized by the largest variation (supplementary fig. S1*C* and *D*, Supplementary Material online). Hotspots of clade diversity are located at the Agulhas Current and in upwelling sites such as the Benguela, California, and Humboldt Currents for both Di*AMT1* and Di*NRT2*, as well as in Antarctic stations where, in particular, Di*AMT1* richness displays a peak (supplementary fig. S1*C*, Supplementary Material online). Clade diversities based on the two gene families follow very similar patterns. The richness of both genes strongly correlate both at surface (SRF, rho = 0.72, *P* < 0.0001) and at deep chlorophyll maximum (DCM, rho = 0.58, *P* < 0.0001). The slopes of the correlation curves indicate that the Di*AMT1*/Di*NRT2* clade richness ratio is substantially constant in SRF (1.18), with Di*AMT1* outnumbering Di*NRT2.* This same ratio increases at the DCM with a higher number of Di*AMT1* with respect to Di*NRT2* (*R_NRT2_* =1.5 + 0.6 *R_AMT1_*). This indicates that a larger suite of solutions is expressed for Di*AMT1* than for Di*NRT2* at the DCM.

Overall, some clades are widespread, thus not showing any specific dependence on environmental conditions, while others seem highly specialized, being related to specific geographic regions and, therefore, to specific conditions (supplementary table S1, Supplementary Material online).

### Both Clades’ Distribution and Abundance Follow an Environmental-Driven Biogeography

**Fig. 2.**
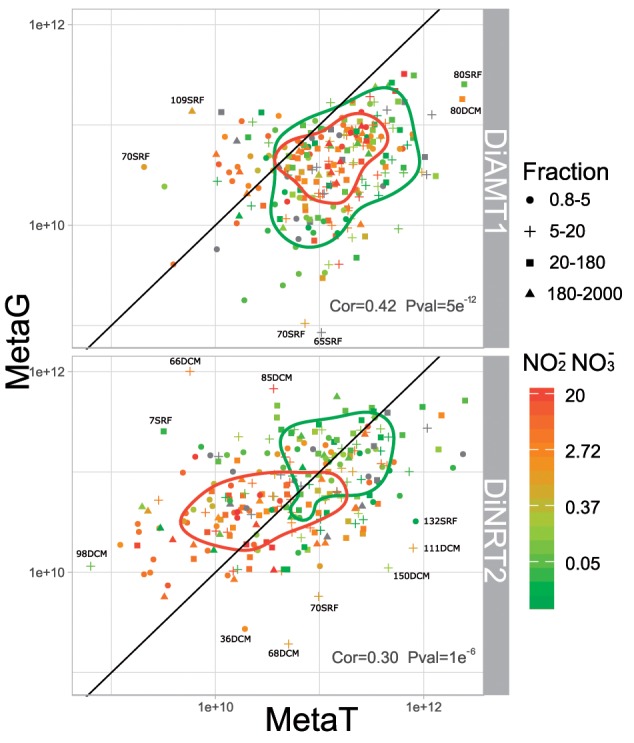
Scatterplot of the occurrences of Di*AMT1* and Di*NRT2* in the metatranscriptome and metagenome data set. Each dot corresponds to a sampling station. They are shaped according to the size class (0.8–5 µm; 5–20 µm; 20–180 µm; 180–2,000 µm) and colored according to the ${\text{NO}}_{2}^{\text{−}}$+${\text{NO}}_{3}^{\text{−}}$ concentration as measured *in situ*. Their ordination in the space is given by the sum of the N transporter unigenes mRNA levels on the x axis and on the sum of the same unigenes abundances in the metagenome on the y axis. A 2-d kernel density estimations of low (<0.4) and high (≥0.4) levels of ${\text{NO}}_{2}^{\text{−}}$+${\text{NO}}_{3}^{\text{−}}$ concentrations (µmol/l) are plotted here as contours. Few stations of interest have been labeled with the sampling station number followed by the sampling depth (i.e., SRF for surface and DCM for deep chlorophyll maximum depth). The Pearson correlation rho and P value have been annotated per gene-family. The correlations are poor and this is due to the behavior of some specific stations acting as outliers. For example, stations 66 and 75 show cases of high DNA abundance and very low mRNA levels, indicating low expression by abundant diatoms. Conversely, the opposite is detected for other stations (e.g., stations 65 and 70), indicating high mRNA levels.

Overall, Di*AMT1* and Di*NRT2* mRNA levels show poor correlation with the corresponding metagenome data (Carradec et al. 2018; fig. 2). Interestingly, highest correlations are found in larger size fractions (20–2,000 µm) indicating a greater stability of the metatranscriptome: metagenome ratio for these diatoms (supplementary fig. S2*A*, Supplementary Material online). Moreover, it is likely that smaller diatoms will tend to have more compact genomes than larger diatoms and, consequently, lower copy numbers (Connolly et al. 2008). This may suggest a higher investment in transcriptional regulation by smaller diatoms: a difference likely contributing to the divergence observed between size fractions. Relative Di*NRT2* metatranscriptome and metagenome occurrences reach very high values when NO_2_^−^+NO_3_^−^ concentrations are low (fig. 2). Even if, Di*AMT1* occurrences are apparently less related to these nutrients (fig. 2), there actually is a size-dependent response, with smaller diatoms *DiAMT1* actually exhibiting low metagenomic occurrences at low NO_2_^−^+NO_3_^−^ concentrations (supplementary fig. S2*A*, Supplementary Material online).

**Fig. 3.**
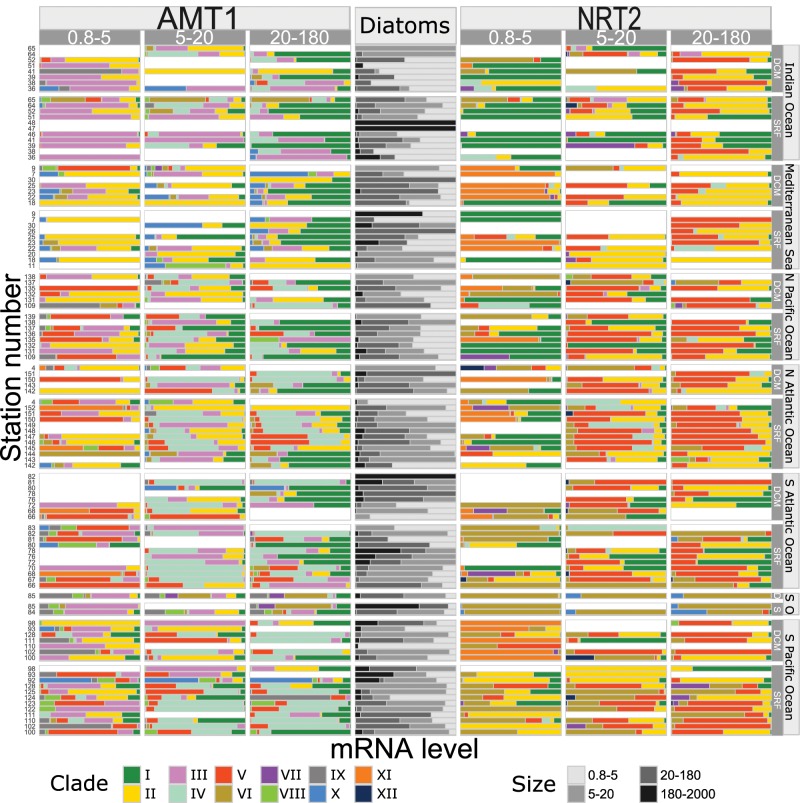
Barplot of clades relative mRNA abundances. Di*AMT1* (columns 1–3) and Di*NRT2* (columns 5–7) clades are showed as distributed across the three size classes (0.8–5, 5–20, and 20–180 μm). Sampling stations are clustered according to the sampling depth, surface (SRF or S) or deep chlorophyll maximum (DCM or D), and oceanic basin (where SO equals to Southern Ocean). In the center column the barplot of relative mRNA abundances of transcripts annotated as diatoms in the different size fractions of the metatranscriptome.

At the clade level (supplementary fig. S2*B*, Supplementary Material online), we observe very different metatranscriptome over metagenome occurrences. Generally, Di*AMT1* clades appear to be more expressed than Di*NRT2* clades, and display narrower ranges of variations in the metatranscriptome: metagenome ratios. Nonetheless, clear differences emerge in the differential expression of Di*AMT1* clades (supplementary fig. S2*B* and supplementary text S1, Supplementary Material online). Generally, group A seems to include genes with a considerable regulation range, displaying higher abundances in metatranscriptome than metagenome data, but also globally high mRNA levels (supplementary fig. S2*B*, Supplementary Material online and fig. 3). By contrast, group B seems to include lowly modulated genes with the median mRNA: DNA occurrences ratio <1 (supplementary fig. S2*B*, Supplementary Material online). Concerning Di*NRT2*, clades prevalently found in centric diatoms (fig. 1) such as clades II, IX, and XII show their copy number exceed mRNA levels in the majority of stations (supplementary fig. S2*B*, Supplementary Material online). Remarkably, similar patterns are observed for clades highly taxonomically affiliated to raphid pennates such as clade I and VI (clade I: 85.6%, clade VI: 41.3%; fig. 1 and supplementary fig. S2*B*, Supplementary Material online).

Geographical distributions of Di*AMT1* and Di*NRT2* clades display a strong regional dependence (supplementary text S2, Supplementary Material online) but also different size-class preferences (fig. 3 and supplementary table S1, Supplementary Material online). For example, clades Di*AMT1*-III and Di*NRT2*–I are predominantly found in communities dominated by small diatoms (fig. 3). It must not be given for granted that size-fractions reflect the diatoms cell size because of spines and chains. According to Malviya et al. (2016)*Tara* Oceans small size fractions (0.8–5 and 5–20 µm) are actually made mostly of small diatoms and single cells. In this study, nanoplanktonic diatoms diverged from larger fraction diatom profiles in terms of both relative mRNA levels (fig. 3) and clade specificity (fig. 3 and supplementary table S1, Supplementary Material online). This is coherent with the different ecological strategies of large and small diatoms toward nutrient uptake (van Oostende et al. 2017).

To analyze the co-occurrence of clades and link it to environmental conditions, we clustered *Tara* Oceans stations on the basis of Di*AMT1* and Di*NRT2* clades presence–absence data and first mapped the clusters geographically and then projected the clusters on the plane of the first two components of an environmental principal component analysis (PCA; supplementary figs. S3 and S4, Supplementary Material online). The spatial distribution of clusters mostly replicates the metabarcode-based biogeography recently published for diatoms (Malviya et al. 2016; supplementary fig. S4*A* and *B*, Supplementary Material online). The Di*AMT1* clustering better discriminates different environmental conditions: it separates areas with relatively high iron concentrations, such as MS and West Atlantic Ocean (Di*AMT1*-*blue*), from the nutrient-limited tropical (Di*AMT1*-*pink*) and other oligotrophic areas (Di*AMT1*-*green*) (supplementary fig. S4*A*, Supplementary Material online). By contrast, Di*NRT2* clustering depicts a wide cluster of stations, Di*NRT2*-*yellow*, widespread throughout all the Atlantic and Mediterranean stations and smaller clusters confined to other specific regions (supplementary fig. S4*B*, Supplementary Material online). Both clusterings (supplementary fig. S3, Supplementary Material online) are mostly explained by N related parameters (in situ NO_3_^−^ and NO_2_^−^), but the relevance of monthly averaged PAR and in situ temperature on one side and modeled iron and in situ NO_3_^−^ on the other suggests the biogeographic role of latitudinal gradients and local dynamics, respectively (supplementary fig. S4*C* and *D*, Supplementary Material online).

Although the complexity of the Di*AMT1* and Di*NRT2* abundance and distribution patterns do not allow to draw a detailed scenario of how N transporters are tuned in the contemporary ocean, all the above suggests that, through evolution, clades differentially evolved N transporters expression toward either constitutive adaptation or specific inducibility to environmental conditions.

### N Transporters Show Differential Responses along the Water Column

Other than geographical gradients, phytoplankton communities have to face great environmental variations along the water column as well. In stratified conditions, the nitrocline acts as a boundary between phytoplankton assimilation, dominating in surface, and microbial oxidation of ${\text{NH}}_{4}^{\text{+}}$, prevailing below the DCM. This duality is due to the different N sources available in the two layers, with ${\text{NH}}_{4}^{\text{+}}$ utilizers above the DCM and NO_3_^−^ utilizers at the DCM (Wan et al. 2018). The correlation of the community distance between the two depths and the environmental parameters (supplementary table S2, Supplementary Material online) showed the Di*AMT1* vertical gradients to be positively correlated with light and ${\text{NH}}_{4}^{\text{+}}$ concentration at the surface. We calculated the ratio of Di*AMT1* over Di*NRT2* mRNA levels at the two sampling depths (supplementary fig. S4*E*, Supplementary Material online). The result shows for small diatoms (0.8–5 µm) a ratio very low in oligotrophic regions and very high in higher NO_3_^−^ and silicate availability regions. This bimodal pattern is partially lost in larger size fractions diatoms, confirming again a size-class dependence in N uptake (van Oostende et al. 2017). Overall, the ratio at surface was significantly lower than the ratio at the DCM (*P* value = 4.2×10^−12^) indicating that Di*AMT1* genes are relatively more abundant than Di*NRT2* at the DCM compared with the surface. An explanation would be diatoms’ rapid exploitation of recycled N in high nutrient availability conditions. This hypothesis could be considered contradictory with the evidence that diatoms are the best utilizers of oxidized N, unless we hypothesize that prokaryotic involvement in NO_3_^−^ assimilation is negligible in the DCM.

**Fig. 4.**
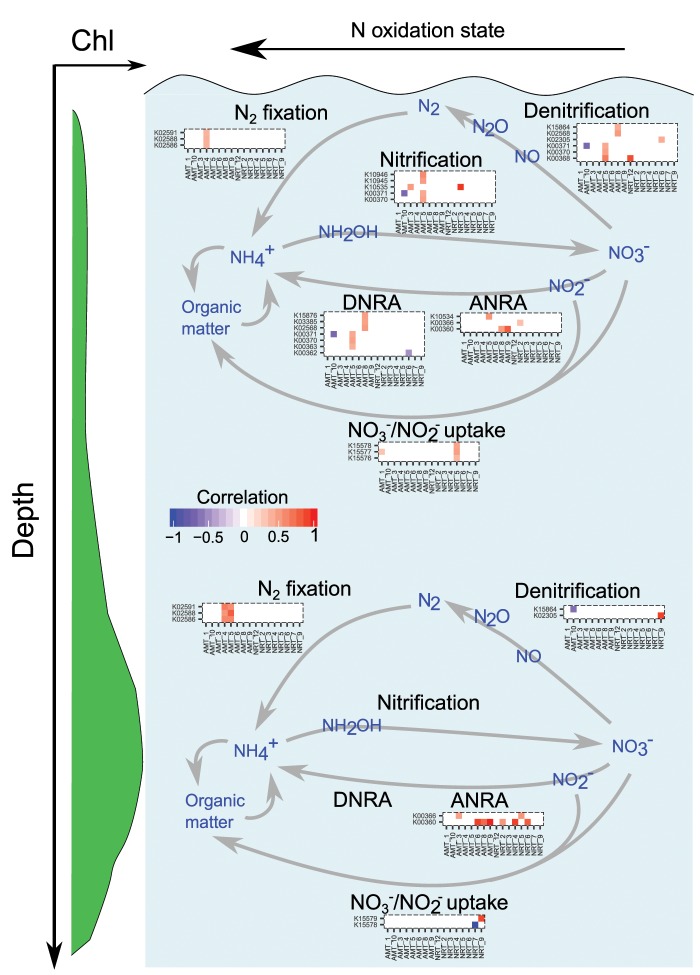
Correlation of diatom Di*AMT1*/Di*NRT2* mRNA abundances against prokaryotic N metabolism gene abundances. The color code indicates the Pearson coefficient for those statistically significant correlations (P value < 0.05). While diatom N transporter abundances are retrieved from the 20–180 µm size fraction, prokaryotic data are obtained from the 0.22–1.6/3 µm ones. Most of the significant correlations have been detected on the Di*AMT1* clades and at surface, suggesting a closer relationship between NH_4_^+^ transporters and prokaryotic modules at this specific depth. Abbreviations: DNRA, dissimilatory nitrate reduction to NH_4_^+^; ANRA, assimilatory nitrate reduction to NH_4_^+^. The enzyme names and definitions for the KEGG orthologous groups (KO) are displayed in supplementary table S3, Supplementary Material online.

We investigated this possibility by analyzing the differential abundance of prokaryotic N metabolism genes at surface and DCM (fig. 4). Comparison of the resulting correlation matrices (fig. 4) indicates that more prokaryotic genes correlate with diatom clades at SRF with respect to DCM, suggesting a tighter compartmentalization between diatom and prokaryote N utilization at surface than at DCM. Very few matches are coherent between the two sampling depths: they are all related to Di*AMT1* clades and linked to prokaryotic processes producing ${\text{NH}}_{4}^{\text{+}}$ such as N_2_ fixation, assimilatory NO_3_^−^ reduction to ${\text{NH}}_{4}^{\text{+}}$ and dissimilatory NO_3_^−^ reduction to ${\text{NH}}_{4}^{\text{+}}$. This depth-independent behavior may be justified by the use of public goods (Olofsson et al. 2019), that is, where high numbers of prokaryotes are present to produce ${\text{NH}}_{4}^{\text{+}}$, transporter clades for uptake of the same substrate are more abundant (Di*AMT1* clades IV, VIII, and IX).

### At the Gene Family Level N Transporters Are Differently Impacted by N Availability

**Fig. 5.**
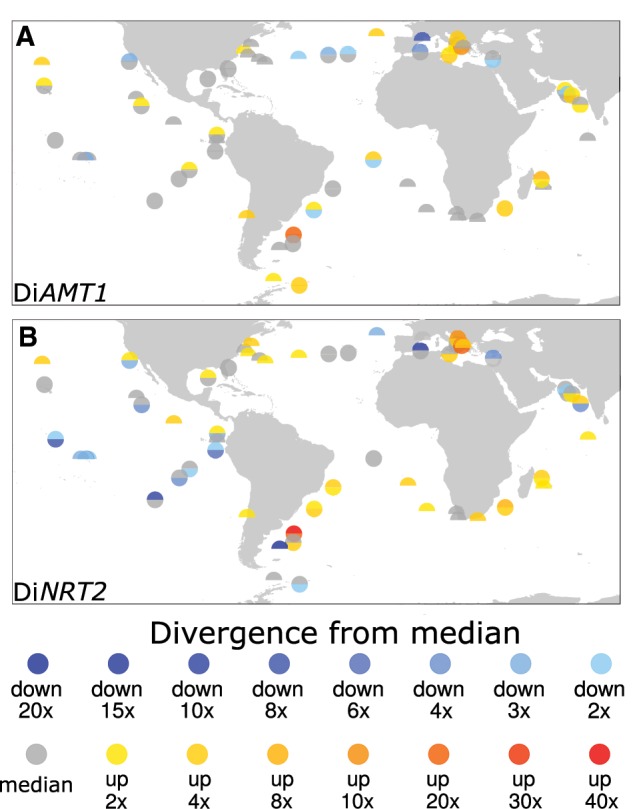
Deviance of mRNA levels of Di*AMT1* (*A*) and Di*NRT2* (*B*) in the size class 20–180 μm. The sum of transcripts assigned to each gene family present at every site is here expressed as fold-change over the median mRNA abundance of the same gene family over the whole *Tara* Oceans data set. Each circle corresponds to a sampling site, while the upper semicircle is filled with the surface value the lower semicircle is filled with the deep chlorophyll maximum information where it is available.

The biogeography exercise previously presented (supplementary figs. S3 and S4, Supplementary Material online) revealed the possible role of the environment on Di*AMT1* and Di*NRT2* regulation. The transcriptional modulation of N transporters (i.e., the deviance from the median abundance; fig. 5) has never been characterized in situ. Among the external factors proved to have a role in this process by in vitro studies there is not only N availability but also light (Bender et al. 2014), Si availability (Smith et al. 2016) and *P* availability (Alexander et al. 2015).

The general pattern of modulation herein observed (fig. 5) shows relatively poor correspondence between the two gene families, suggesting a complex, likely compensating, regulatory system. In the tropical Pacific and Antarctic stations Di*AMT1* mRNA levels diverged only slightly from the median, whereas Di*NRT2* mRNA abundances are low. Both Di*AMT1* and Di*NRT2* are abundant in areas of low N availability (e.g., MS and IO). By contrast, in conditions of high N availability, Di*NRT2*s show low mRNA levels while Di*AMT1* are not differentially modulated. This may be due to the differential location of Di*NRT2* genes in the cell and the storage of N in replete conditions. These regions, such as the Antarctic stations and the Humboldt current upwelling sites, are indeed characterized by a higher use of putative vacuolar (VM) Di*NRT2*s rather than plasma membrane (PM) Di*NRT2* (supplementary fig. S5, Supplementary Material online), at least in the small- and medium-sized diatoms (0.8–180 µm).

The different geographical patterns are mirrored by the strongly different results of mRNA levels correlations with environmental variables (supplementary table S2, Supplementary Material online). Di*NRT2* transcripts anticorrelate significantly in surface and at DCM with in situ measured NO_2_^−^, NO_2_^−^+ NO_3_^−^, NO_3_^−^, ${\text{PO}}_{4}^{3}$^−^, Si and modeled ${\text{NH}}_{4}^{\text{+}}$, while it is positively correlated with modeled iron availability. Di*AMT1* transcripts are inversely related to latitude and temperature, indicating a regionalization of Di*AMT1* mRNA levels, but it is also positively correlated to in situ measured silica.

### Temperature, Iron, and Substrate Availability Modulate Di*AMT1* and Di*NRT2* Clades Expression in the Ocean

To better understand the fine response of N transporters to the different environmental drivers, we investigated the modulation of single phylogenetic clades. Interestingly, mRNA levels of Di*AMT1* and Di*NRT2* clades are correlated negatively with N related variables (supplementary fig. S6, Supplementary Material online). This may indicate that, independently of other N sources, the major response to NO_3_^−^ NO_2_^−^ replete conditions for several clades is to decrease the abundance of mRNAs encoding the N uptake machinery. By contrast, specific clades such as Di*AMT1* clades IV and V, that we already suggested being modulated by ${\text{NH}}_{4}^{\text{+}}$ availability (fig. 4), do show either positive or no correlations at all with NO_3_^−^ NO_2_^−^ availability, corroborating the previous hypothesis. Moreover, another clear exception is seen for Di*NRT2* clade VI, which is strongly positively correlated with different sources of N availability. This could be driven by the role of the genes involved in N storage included in this clade.

**Fig. 6.**
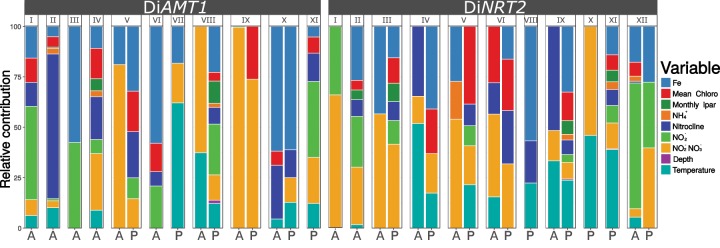
Relative contributions of the nine environmental predictors selected for the BRT modeling. Contribution is expressed as number of times a variable is selected for splitting the regression trees branches, weighted by the squared improvement to the model as a result of each split, averaged over all trees and scaled to 100. This information is expressed both for models based on presence–absence (P) and on mRNA abundance data on the 20–180 µm size fraction (A).

To better test whether and, if so, which environmental variables trigger the expression of N transporter genes, we applied the Boosted Regression Tree method (BRT) (Elith et al. 2008; fig. 6). This machine learning technique has been proposed to delineate the niche of a group of organisms, identifying the best predictor variables for a given event and taking into account nonlinear relationships between the variables. Herein, we link both presence–absence (the “niche”) and abundance (the mRNA level) data of the N transporter clades to the environmental variables. Analyses were restricted to the 20–180 µm size fraction as it contains the higher abundance of diatoms in the *Tara* Oceans data set (Malviya et al. 2016; supplementary fig. S8, Supplementary Material online). For both gene families, iron and NO_3_^−^ were found to play a leading role in defining clade distributions, while temperature was also important for Di*NRT2* (fig. 6 and supplementary fig. S7*A*, Supplementary Material online). Another strong contributor is chlorophyll α, proxy of biomass accumulation, suggesting a possible link between growth rates and the specifics of N uptake. Concerning mRNA abundances, iron and NO_2_^−^NO_3_^−^ were by far the most important contributors for Di*AMT1* transporters (supplementary fig. S7*A*, Supplementary Material online) while Di*NRT2* mRNA levels were also related to nitrocline depth and NO_2_^−^. Clade-level mRNA levels seem to be preferentially controlled by N availability, even more than the corresponding models for presence–absence. Of note, mRNA levels may be modulated also by the availability of different N sources as some Di*AMT1* clades (clades I, III, and VI) are more regulated by NO_2_^−^, while others (clades V, VIII, and IX) were mostly explained by NO_3_^−^ availability. Peculiarly, Di*AMT1* clade XI shows no sign of environment specificity (fig. 5). It is thus possible that the emergence of this ancestrally diverging polar-centric-Mediophyceae clade may reflect functional redundancy in this diatom class.

**Fig. 7.**
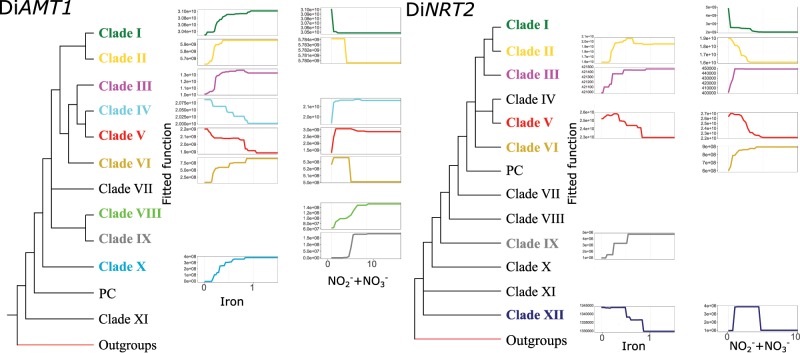
Response curves derived from the BRT models. Herein are showed the univariate response curves from models based on mRNA abundances and only for the two most contributing variables of the models: iron and ${\text{NO}}_{3}^{\text{−}}$+${\text{NO}}_{2}^{\text{−}}$ concentrations. The iron concentrations is expressed in nmol/l while ${\text{NO}}_{2}^{\text{−}}$+${\text{NO}}_{3}^{\text{−}}$ concentration is expressed in µmol/l. Curves are colored as the clades they refer to, depicted on the stylized phylogenetic tree on the left.

While the contributions (fig. 6 and supplementary fig. S7*A*, Supplementary Material online) reflect the relevance of the variables for the models, the response curves (fig. 7) show the assessed univariate niches of the clades. Taking into account the two variables showing the highest contribution through the models (iron and NO_3_^−^ concentrations; fig. 7), it is worth noting that the Di*AMT1* clades absent in pennate diatoms (namely, clades I and II), show clear distinguishable response curves (fig. 7). Surprisingly, these are slightly similar to Di*AMT1* clade VI ones too which, even if belonging to pennates, shows a very peculiar distribution likely transcriptionally adapted to cold conditions. Clade VII, which is basal to group A, is specifically influenced by temperature, being strictly located in Antarctic stations (fig. 6) (the link with temperature is discussed in more detail below). Di*NRT2* clade-based response curves (fig. 7) are suggestive of a specific response to NO_3_^−^ displayed by generalist clades, that is, clades belonging to at least three out of four diatom classes (namely, clades I, II, and V). Interestingly, clade VI, while found in all diatom classes, shows a similar response to clade III (raphid pennates). This may be explained by the fact that the transcriptomic scenario of clade VI is indeed dominated by raphid pennates, with other classes giving only a minor contribution. The basal clade XII shows a very high contribution of NO_2_^−^ concentration to modeled mRNA abundance, which indicates that ancestral Di*NRT2* mRNA abundance was specifically dependent on the substrate (fig. 6).

BRT-based analysis further highlighted a clear dependence of clade presence–absence on temperature, which resulted a significant variable for 10 abundance clade models over 15 (fig. 6 and supplementary fig. S7*A*, Supplementary Material online). Because a global increase of sea water temperature is predicted in the present scenario of climate change, we used the models to investigate the possible impact of ocean warming on diatom N uptake by projecting changes in the clades more or less affected by temperature in a future ocean. We computed the probability of the presence of each clade in every sampled station with an increase of temperature up to 3.0 °C, with 0.5 °C increments, maintaining all the remaining variables fixed to the observed values. Di*NRT2* clades were the most temperature-sensitive (supplementary fig. S7*B*, Supplementary Material online), showing three clades with strongly narrower distributions caused by increased temperatures (clades IX, X, and XI). Because these three clades are all basal to supergroup A Di*NRT2*, one may speculate that the primitive Di*NRT2* was not preferentially adapted to high temperature. Within the Di*AMT1* family, clade VII is the most negatively affected by temperature increases and as expected by the current distribution in Antarctic regions.

## Discussion

In this study, we have explored the potential of marine meta-omics for the in-depth analysis of a functional trait in diatoms. Our working hypothesis was that N transporter gene evolution partially predates adaptive evolution in diatom N uptake. Consequently, we expected to observe a differential usage of evolutionary solutions across biogeography patterns and vertical depth, all induced by environmentally driven regulation. Although the lack of resolution of our data set does not allow us to perform a fine-scale analysis of gene modulation, our results partially support this hypothesis.

The recently published *Tara* Oceans global eukaryotic metatranscriptome (Carradec et al. 2018) gave us access to an unprecedented amount of in situ data. Evolutionary relationships of diatom high affinity N transporters have been inferred using a data set that largely expands previous ones (Rogato et al. 2015), generating significant new information concerning diatom transporters diversification. Both *AMT1* and *NRT2* originated prior to diatom emergence and they share common molecular signatures with other autotrophic marine and land organisms (von Wittgenstein et al. 2014). Nonetheless, within the diatoms both gene families show specific evolutionary patterns. The complex evolutionary scenario reconstructed is strongly affected by gene loss and duplications. While the Di*AMT1* family shows clade reduction in Bacillariophytina (and especially in araphid and raphid pennates), Di*NRT2* shows family expansion in the same lineage (especially in Mediophyceae), with some gene loss in pennate diatoms. It has been recently demonstrated that a key event having a major impact in diatom radiation was subduction of the Tethyan Trench (ca. 150 ma) (Lewitus et al. 2018). This is compatible with the divergence of pennate diatoms from Mediophyceae diatoms within the Bacillariophytina lineage (Medlin 2016). Indeed, it is thus possible that the increased diversity in diatom species resulted in a rearrangement of the repertoire of high-affinity transporters in response to the high nutrient influx related to this event (Lewitus et al. 2018).

Subcellular localization of the proteins may be one of the main drivers of both evolution and functional diversity in Di*NRT2*. Caution is required as the predicted subcellular localization of the Di*NRT2* is not experimentally validated but it is extremely intriguing the higher percentage of vacuolar genes found in diatoms compared with vascular plants (see supplementary text S3, Supplementary Material online). This may be explained by the evolutionary advantage of NO_3_^−^ storage in diatoms, which live in a highly changing environment, favoring vacuolar Di*NRT2* duplication.

Previous reports (i.e., Rogato et al. 2015) indicate that modulation of N transporter expression is based on many different factors and it is far from being fully understood. Interestingly for Di*NRT2*, we found that not only the metatranscriptomic but also the metagenomic abundances reflected substrate availabilities, with particularly higher abundances of both in low NO_3_^−^ concentrations. Our results suggest that N transporter evolution led to a differentiation between genes able to feature environmentally driven acclimatization and genes which are not. In the first case, the observed metatranscriptomic pattern is shaped by both copy number and mRNA levels, in the latter case it is more related to variations in the abundance of taxa and genes (ecological turnover—Salazar and Sunagawa 2017). An example of the first case is herein given by Di*AMT1* clade VI, which shows high constitutive mRNA levels in low temperature conditions, suggesting adaptation of the species to these conditions. To note, other mechanisms of regulation such as posttranslation regulations (in particular, phosphorylation mechanisms) have been reported for both N transporters (Jacquot et al. 2017), however these mechanisms cannot be investigated through metatranscriptomic and metagenomic studies. As expected (van Oostende et al. 2017), a size-class effect was detected in the differential use of different N transporters between small and medium/large diatoms, highlighting different approaches to N uptake.

Overall, we found that diatoms locally deploy a more extensive repertoire of genetic solutions (clade richness) for ${\text{NH}}_{4}^{\text{+}}$ uptake compared with NO_3_^−^. This is also reflected by the fact that the mRNA abundances of 3 over 12 Di*NRT2* clades are globally dominant, while Di*AMT1* transcript abundances are widely divergent across clades and regions. This likely indicates that diatom AMT1 proteins are more specialized to local conditions, while functional redundancy may be dominant in DiNRT2. Among the possible evolved physiological solutions, a differential expression of the Di*AMT1* genes in response to a tight compartmentalization with ${\text{NH}}_{4}^{\text{+}}$ producing prokaryotes must be taken in consideration (Olofsson et al. 2019; fig. 4). Such a “good neighborly” context in ocean environments, might recall the one observed between land plants and root-associated microbiota, where a cross-talk between bacteria-based ${\text{NH}}_{4}^{\text{+}}$ producing and plant-based ${\text{NH}}_{4}^{\text{+}}$ assimilation pathways has been identified (Becker et al. 2002; van Deynze et al. 2018).

A novelty of the present work is represented by the use of machine learning techniques like the BRT approach to model the multivariate space of Di*AMT1* and Di*NRT2*. This approach enabled us not only to establish the contribution of the different environmental variables in defying the presence and usage of these two gene families but also to predict their future behavior in the frame of global warming. This analysis does not claim to realistically predict future scenarios as only the temperature variable was taken into account. Indeed, high quality future forecasts of other key parameters are required to reliably predict the evolution of diatom functionality. In particular, the most contributing variables emerged from the BRT analysis, such as iron or NO_3_^−^, may be essential for this purpose.

To conclude, a remarkable complexity of evolutionary solutions and gene expression regulation emerged from this study, highlighting the sophisticated behaviors of diatoms as a group. This finding undermines the general view of diatoms responding uniformly to nitrogen, especially NO_3_^−^, availability and advocates for the need of a deeper understanding of the factors that concur in the regulation of the uptake of all forms of nitrogen along with the different environmental contexts, likely contributing to their ecological and functional differentiation.

## Materials and Methods

### Di*AMT1* and Di*NRT2* Identification in the *Tara* Oceans Eukaryote Unigenes Catalog

Extensive search for putative Di*AMT2* genes was performed in both the MATOU (Alberti et al. 2017; Carradec et al. 2018) and the MMETSP (Keeling et al. 2014) databases, using the TBlastN program. Given the absence of diatom *AMT2* homologues in the reference literature, *AMT2* homologues from a green plant (*Arabidopsis thaliana*) and from a coccolithophore (*Emiliania huxleyi*) were used to perform the Blast search. MATOU is a catalog of 116 million unigenes obtained from poly-A+ cDNA sequencing of different filter size fractions ranging from 0.8 to 2,000 μm (Carradec et al. 2018), representing the largest reference collection of eukaryotic transcripts from any single biome. Here, we analyzed a total of 107 samples from 65 globally distributed stations including surface (n = 65) and DCM (n = 42) seawater samples from four different size fractions (0.8–5, 5–20, 20–180, and 180–2,000 μm). The geographical distribution of the 65 *Tara* Oceans sampling stations is represented in supplementary figure S8, Supplementary Material online.

Di*AMT1* and Di*NRT2* sequences from a previous report (Rogato et al. 2015) were used as queries to search against the Marine Atlas of *Tara* Oceans Unigenes (MATOU) Database (Alberti et al. 2017; Carradec et al. 2018) by TBlastN program (Gertz et al. 2006). Search was performed through CLADE, which predicted a domain architecture for each reference sequence (Bernardes et al. 2016). Profile HMMs corresponding to the detected domain architectures (available in Pfam database) were saved into an initial pHMM database. This database was enriched by adding three new pHMMs: one built from the entire set of reference sequences, and two others built exclusively from diatoms and nondiatoms reference sequences. The pHMM database was then used to scan the six frame translations of *Tara* Oceans metatranscriptome data set. For that, we used HMMer version 3.1 (with -cut_ga option) and detected more than 6,000 *Tara* Oceans sequences as the referred transporters. To reduce false positives, a second run of CLADE over the 6,000 *Tara* sequences (all six frame translations) was performed to produce the most probable domain architecture for each sequence. We analyzed these domain architectures and only sequences containing at least one domains of pHMM database were considered (10% of the initial set of sequences). To select the most probable translation, DAMA (Bernardes et al. 2016) was modified to consider the six frames. We consider as putative transporter only the most probable frame translation that present the same domain architecture of the reference sequences. The putative transporters were then split into diatom and nondiatom species. For that, we checked the taxon agreement of pHMM model, and of CLADE results. Only sequences with diatom taxon on both models were considered to be true diatoms transporters. 529 putative Di*AMT* and 471 putative Di*NRT2* sequences were retrieved from the search. The obtained taxonomic assignation of Di*AMT* and Di*NRT2* sequences was compared with the one obtained by blasting the sequences against the MMETSP using an in-house developed Blast tool. Sequences were thus assigned selecting the best value (supplementary data files S3 and S4, Supplementary Material online).

### DiAMT1 and DiNRT2 Alignment

The reference set of *AMT1* and *NRT2* sequences from Rogato et al. (2015) was used as query against the 92 diatom transcriptomes from MMETSP (Keeling et al. 2014) using an in-house developed pBLAST search. 45 Di*AMT1* and 51 Di*NRT2* sequences were obtained in this step. A multiple sequence alignment was carried out with Muscle (Edgar 2004) for a set of translated nucleotic sequences of both Di*AMT1* and Di*NRT2*, including the diatoms sequences retrieved from *Tara* Oceans and MMETSP projects plus appropriate nondiatoms sequences. Two trimmed alignment were obtained (supplementary data files S7 and S8, Supplementary Material online). The Di*AMT1* alignment is composed of 282 sequences, of which 162 corresponds to the *Tara* Oceans eukaryotic catalog, and consists of 137 AA positions (including gaps). The Di*NRT2* alignment includes 259 AA sequences (166 from the *Tara* Oceans eukaryotic data set) and consists of 108 positions (including gaps). Consensus sequences for the Di*AMT1* and Di*NRT2* alignments were graphically represented using sequence logo at the Web Logo website (http://weblogo.berkeley.edu/logo.cgi).

### DiAMT1 and DiNRT2 Phylogenies

Phylogenetic analyses were performed using 1) approximately maximum-likelihood method (aML—Anisimova and Gascuel 2006) and 2) Bayesian Inference (BI—Mau et al. 1999) approaches. To infer the aML phylogenetic relationships, we used the FastTree2 software (Price et al. 2010). The BI analysis was conducted using MRBAYES v3.2 (Ronquist et al. 2012). Trees were sampled every 1,000 generations for six million generations, and the first 25% of all the trees sampled were discarded as burn-in. From the phylogenies were manually defined 11 clades for the Di*AMT1* and 12 for the Di*NRT2*.

### Data Mining and Normalization

The presence–absence of each clade for both families is defined by their detection in the metatranscriptome database of *Tara* Oceans. A clade is considered present in a sampling site if their mRNA abundance is >0 in at least one of the four size classes sampled (0.8–5, 5–20, 20–180, and 180–2,000 μm). In terms of mRNA values, occurrences are computed per size class in the metatranscriptome data set as fraction of number of reads mapped per kb of transporter gene covered with reads per the total number of reads mapped to diatoms in that sample, in terms of DNA values, occurrences are computed with the same procedure in the metagenomic data set. The total number of reads mapped to diatoms per metatranscriptomic and metagenomic sample can be found in supplementary data file S10, Supplementary Material online.

### Metatranscriptome and Metagenome Comparison

The normalized mRNA abundance per family was compared with the corresponding DNA through a Pearson correlation, on both all size fraction data together and on the subsets of different size fractions. A ratio of the mRNA and DNA abundance per clade has been compared with the in situ measurement of NO_2_^−^ NO_3_^−^ (Pesant et al. 2015; PANGAEA doi: 10.1594/PANGAEA.836319).

### DiAMT1 and DiNRT2 Clade Distribution

Zero-adjusted Sørensen dissimilarity coefficient (Clarke et al. 2006) were computed for the 106 *Tara* Oceans stations on both gene families’ presence–absence data. Stations have been clustered applying the Ward’s minimum variance method (Murtagh and Legendre 2014). The clustering method choice as well as the optimal cutting level value were supported by the silhouette width of the observations (supplementary fig. S3, Supplementary Material online). A number of eight and nine clusters of stations were defined, respectively, for Di*AMT1* and Di*NRT2* clades over their presence–absence.

### Selection of Environmental Parameters

The environmental descriptors were selected to be key variables a priori related to diatoms N transporter and uncorrelated between them (Pearson correlation coefficient <0.6). The following nine environmental parameters were chosen: 1) Mean chlorophyll α (mg/m^3^) measured in situ (Pesant et al. 2015; PANGAEA doi: 10.1594/PANGAEA.836321), 2) Mean monthly iron concentration (nmol/l), extracted by the PISCES2 (Aumont et al. 2015) model, 3) Monthly average PAR, based on satellite data, 4) Annual mean of surface ${\text{NH}}_{4}^{\text{+}}$ concentration, extracted by World Ocean Atlas 13 (Boyer et al. 2013), 5) NO_2_^−^ NO_3_^−^ concentration (μmol/l), as measured in situ (Pesant et al. 2015; PANGAEA doi: 10.1594/PANGAEA.836319), 6) NO_3_^−^ concentration (μmol/l) as measured in situ (Pesant et al. 2015; PANGAEA doi: 10.1594/PANGAEA.836319), 7) Temperature (°C), measured in situ (Pesant et al. 2015; PANGAEA doi: 10.1594/PANGAEA.836321), 8) Mean nitrocline depth (m), measured in situ (Pesant et al. 2015; PANGAEA doi: 10.1594/PANGAEA.836321), and 9) Sampling depth, categorical information for surface and DCM samples.

### Prediction of Subcellular Localization

The subcellular localization has been predicted by running the DiNRT2 sequences through the LocTree3 software, PSI-BLAST, pipeline PredictedProtein (supplementary data file S9, Supplementary Material online; Goldberg et al. 2014). The reliability of the LocTree3 software has been tested by confirming the sublocalization (plasma membrane or vacuolar membrane) of 11 plant NRT2 sequences whose localization has been experimentally verified (7 in *A. thaliana*, 3 in *Oriza sativa*, 1 in *Chrysanthemum morifolium*). The complete 19 plant NRT2 family sequences analyzed to compare the distribution of plasma membrane- and vacuole-localized NRT2 proteins are from: *A thaliana*, *Lotus japonicus*, *Medicago truncatula*, *Phaseolus vulgaris*, *Vitis vinifera*, *Malus domestica*, *Glycine max*, *Nicotiana tabacum*, *C morifolium*, *Triticum aestivum*, *Daucus carota*, *Prunus persica*, *Fragaria vesca*, *Solanum tuberosum*, *Hordeum vulgare*, *O sativa*, *Zea mays*, *Solanum lycopersicum*, *Populus trichocarpa*, and *Solanum tuberosum.* The level of significance was tested with the Fisher’s exact probability test.

### Environmental PCA

Principal component analysis was performed on a subset of environmental variables specifically selected for each gene family presence–absence using the *bioenv* function (supplementary data file S11, Supplementary Material online). Euclidean distances were chosen for environmental variables, while Sørensen distances for the clade presence–absence. Stations clade-based clusters were mapped on the PCA biplot through a discrete colorimetric scale.

### Transporter Richness

Transporters richness is expressed for every site as the number of clades detected. The richness of Di*AMT1* and Di*NRT2* has been compared through a Pearson correlation analysis per sampling depth. One-tail *t*-tests have been performed on the richness values to test if this index in surface is higher than at DCM for both gene families.

### mRNA Level Profiles

To compare the mRNA levels of the two gene families, the transcripts mRNA abundance per family was compared with the median of the observed values per gene family. The relationship with environmental parameters of the total transcripts per family was investigated through multiple Spearman correlations with all the environmental variables available (Pesant et al. 2015; PANGAEA doi: 10.1594/PANGAEA.836319 and PANGAEA doi: 10.1594/PANGAEA.836321), with a “fdr” *P* value adjustment. Correlations were considered significant with a *P* value <0.05 and an absolute coefficient >30. Clade mRNA profiles were correlated with environmental parameters with a pairwise Spearman correlations “fdr” adjusted.

### Vertical Switch

The zero-adjusted Bray-Curtis distance (Clarke et al. 2006) between surface and DCM samples on the mRNA levels of Di*AMT1* and Di*NRT2* clades was computed and correlated (Spearman) to the single environmental parameters of both depths, with “fdr” *P* value adjustment. The total sum of transcripts of Di*AMT1* and Di*NRT2* was computed and their ratio in surface over DCM was obtained per each station. A one-tail *t*-test tested the relationship between the two ratios to test if the ratio Di*AMT1*/Di*NRT2* at surface is significantly lower than the same ratio at DCM. We searched for genes involved in prokaryotic N metabolisms in the same *Tara* Oceans stations and depths where transcripts for diatom N transporters were retrieved. For this, we mined the Ocean Microbial Reference Gene Catalog (OM-RGC.v1), a comprehensive collection of 40 million nonredundant genes from mostly free-living prokaryotic communities (Sunagawa et al. 2015). This catalog was functionally annotated using the KEGG database (Kyoto Encyclopedia of Genes and Genomes; https://www.genome.jp/kegg/; last accessed July 5, 2019; Kanehisa et al. 2008) into KEGG orthologous groups (KOs) (e.g., nitrogenase iron protein NifH and nitrite reductase [NADH] large subunit) and KEGG modules (e.g., nitrogen fixation, and denitrification). Therefore, we retrieved the abundance profiles for KOs associated to prokaryotic N metabolisms (size fraction 0.22–1.6/3 μm) from this catalog and we compared them with the diatom clade mRNA abundance profiles for the same geographical site and depth. Specifically, Pearson correlations were computed for each surface and DCM station between the diatom clade mRNA levels and the prokaryotic gene levels associated to N metabolism (Sunagawa et al. 2015).

### Boosted Regression Tree Modeling

BRT models (Elith et al. 2008) were run through the *dismo* and *gbm* R packages (Ridgeway 2006; Hijmans et al. 2017) to model both the presence–absence and the mRNA profiles (20–180 μm) of each clade of the two gene families. Previously selected environmental variables were exploited as predictor variables. Models used Bernoulli and Laplace distributions, slow learning rates (0.001–0.005), tree complexity equal to 5- and 10-fold cross-validated with a 50% bag fraction. Each model was simplified and a k-fold cross-validation procedure was applied to select the optimal number of trees. Statistical significance was assessed through cross validated AUC score (>0.7) for presence–absence based models. The significance of mRNA-based models has been estimated by the significance of the Pearson correlation between the observed and predicted mRNA levels classed in quartiles (*P* value <0.05; |rho| >0.35). The probability of presence–absence of each clade in every sampling stations was predicted in different temperature increase scenarios (up to 3 °C every 0.5 °C). All the BRT statistics can be found in supplementary data file S11, Supplementary Material online. The resulting probabilities were translated in presence–absence applying a MaxSens+Spec threshold computed through the *PresenceAbsence* R package (Freeman and Moisen 2008). From these results the occurrence frequency of each clade in every temperature scenario was calculated as the percentage of stations where the clade is present.

## Supplementary Material

Supplementary data are available at *Molecular Biology and Evolution* online.

## Supplementary Material

msz157_Supplementary_Data
