# Supplementary material for: Meta-Omics Reveals Genetic Flexibility of Diatom Nitrogen Transporters in Response to Environmental Changes
Source: Mol Biol Evol. 2019 Jul 1;36(11):2522–35. doi: 10.1093/molbev/msz157 (PMC6805229; doi:10.1093/molbev/msz157)
Supplement: msz157_Supplementary_Data [file msz157_supplementary_data.zip › Busseni_supp_revised.pdf]

# Supplementary Materials

## Supporting text

### Text S1

**Observations on Di*AMT1* clades mRNA-DNA occurrences.** Clades VIII and IX display high gene and very low mRNA abundances, with few exceptions (stations 80 DCM and 102 SRF, respectively), suggesting the constitutive low expression of these clades, and the requirement of specific conditions for their transcriptional modulation (Fig. S2B). This similar behavior reflect the phylogenetic relationship of these clades. Indeed, clades VIII and IX are sister clades (Fig. 1) and both include *AMT1* found in centric and raphid-pennate diatoms.

### Text S2

**Examples of clades biogeographies.** The ubiquity of recent Di*AMT1* clades I-III (Table S1) hints at a possible wider specialization of the proteins belonging to those clades, which are expressed in different environmental conditions. By contrast, Di*AMT1* clade VI, which is particularly enriched in araphid diatoms genes (Fig. 1), seems to be specifically related to species thriving in low temperature regions, as the polar species *T. antarctica* to which four out of six araphid Di*AMT1*-VI have been annotated (Fig. S1A). Another example is found in the Mediterranean Sea (MS): while clade Di*AMT1*-X is predominantly found here, clades Di*AMT1*-IV and Di*AMT1*-V are present everywhere except the MS, suggesting a recent appearance of this clade (Fig. S3). Di*NRT2* clades as well display some regional dependence, either related to the species utilizing them or to the environmental conditions triggering their expression.

### Text S3

**Nitrate storage in vacuolar plants.** Nitrate loading machinery into the vacuole deals with cytosolic concentration, which may depend by nutrient availability in the environment as well as efficiency of $NO_3^-$ reduction pathways. High capacity of vacuolar $NO_3^-$ accumulation (30-50 mM) and release has been also reported in vascular plants where it plays a physiological role during the dark-to-light transition associated to increased NR activity and it is extremely important for $NO_3^-$ reallocation and efficient utilization when external N supply is limited. In vascular plants the estimated physiological

cytosolic concentration of $NO_3^-$ varies from 1 to 5 mM and the only $NO_3^-$ transporters involved so far in the $NO_3^-$ loading into the vacuole are the A. thaliana AtNRT2.7 regulating the kinetic of seed germination and the chloride channel member AtCLCa, a $NO_3^-/1H^+$ antiporter expressed in shoot and roots, responsible for 50% of $NO_3^-$ accumulation into vacuoles.

**Fig. S1. Conserved regions of DiAMT1 and DiNRT2 protein sequences and global pattern of richness distribution.** WebLogo consensus sequences for DiAMT1 and DiNRT2 are showed in panels (**A**) and (**B**). The LGTF signature of DiAMT1 is located in the 6$^{th}$ TM domain, whereas the DiNRT2 GVELT signature is inside the 7$^{th}$ TM domain. In the DiNRT2 signature, the glutamate residue (E) within the motif is conserved in all eukaryotic nitrate transporters and is likely involved in the formation of salt-bridge with other conserved charged amino acids for stabilization of protein conformation. Richness expressed as the number of phylogenetic clades present in each station based on DiAMT1 (**C**) and DiNRT2 (**D**). For both gene families the maximum number of clades observed in a station is of 10. Each circle corresponds to a sampling site, while the upper semicircle is filled with the surface value the lower semicircle is filled with the deep chlorophyll maximum depth information where it is available.

**Fig. S2. Relationship between the gene and mRNA abundance of the two gene families considered as a whole or as phylogenetic clades.** (**A**) Gene families' metatranscriptomic and metagenomic occurrences across the four different size classes for both Di*AMT1* and Di*NRT2*. Pearson correlations are annotated in each panel. Stations are colored according to the $NO_2^-$+$NO_3^-$ concentration measured *in situ*. (**B**) Boxplot of the ratio of metaT and metaG abundances of Di*AMT1* and Di*NRT2* clades.

**Fig. S3. Di*AMT1* and Di*NRT2* clades presence-absence clustering**. Heatmaps showing Di*AMT1* (**A**) and Di*NRT2* (**B**) clades presence-absence in the *Tara* Oceans stations. Stations are clustered by a Ward clustering method based on zero-adjusted Sørenson dissimilarity between the samples based on clades presence absence and annotated in 8 resulting clusters for Di*AMT1* and 9 resulting cluster for Di*NRT2*. The white cells correspond to the stations where the corresponding clade is absent; the gray cells correspond to the stations where the relative clade has been found. In panels (**C**) and (**D**) are the estimated silhouette values for each established cluster of Di*AMT1* and Di*NRT2* respectively. Values closer to 1 indicate a high degree of similarity of the station within the cluster, positive values close to zero indicate stations which are closer to the other clusters, while negative values indicate stations which may have been misplaced by the clustering. The silhouette defines the clustering as acceptable if all the clusters have elements higher than the average value. The number of sampled stations and average silhouette value for each cluster are displayed in the right margin.

**Fig. S4. Di*AMT1* and Di*NRT2* biogeographies**. Geographical clusters on DiAMT1 (**A**) and DiNRT2 (**B**) clades presence/absence. The top portion of each circle represents samples collected at the surface and the bottom portion represents the deep chlorophyll maximum depth (stations missing metatranscriptome data for one of the two depths are drawn as half circles). The biplots of the environmental PCA of Di*AMT1* (**C**) and Di*NRT2* (**D**) with a density contour of the clusters previously defined. Each point corresponds to a sampled station and the arrows correspond to the descriptors of the PCA space. Eight clusters result from Di*AMT1* data while nine clusters from Di*NRT2*. Clusters are identified by up to nine colors per gene family: 1-yellow, 2-cyan, 3-pink, 4-blue, 5-red, 6-green, 7-orange and 8-dark green and 9-violet, as defined in Fig. S4. In panel (**E**) the ratio between Di*AMT1* mRNA abundance and Di*NRT2* mRNA level computed in surface (top portion of each circle) and at DCM (down portion of each circle). Every panel refers to one of the four size classes taken into account.

**Fig. S5. Map of the ratio of vacuolar Di*NRT2* mRNA abundance over the plasmatic membrane Di*NRT2* mRNA abundance.** The data is shown as measured in the 4 different size classes taken into account. Each circle corresponds to a sampling site, where the upper semicircle is filled with the surface value while the lower semicircle is filled with the deep chlorophyll maximum depth information where it is available.

**Fig. S6. Pairwise Spearman correlation between N transporter clade mRNA level and environmental parameters.** The correlations are run for every size class and fdr adjusted. Only significant correlations are shown (p-value $<0.05$), and the number of samples on which the correlation is computed is written in the corresponding cell.

**Fig. S7. BRT contribution and prediction outputs.** (**A**) Boxplot of the contribution of environmental predictors in detecting clades optimal conditions, from the BRT models based on both presence-absence (P) and mRNA abundance (A). (**B**) Sensitivity exercise on clades BRT models to the temperature variable. Prediction of ubiquity changes on the *Tara* Oceans sampling stations varying only the temperature parameter up to 3.0°C every 0.5°C. Ubiquity is expressed in percentage as the change of number of stations where each clade is present in relation to the occupancy observed in the real data expressed as 100%.

**Fig. S8. *Tara* Oceans sampling stations.** Geographic distribution of the 65 *Tara* Oceans sampling stations at which seawater samples and environmental data were collected from surface and deep chlorophyll maximum depths.  The color code refers to the oceanographic basin they are located in.

**Tab. S1. Synthesis of the information collected on Di*AMT1* and Di*NRT2* clades.** Information extracted from phylogenetic, biogeographical and expression modulation analyses are here summarized. For each clade it is described the taxonomic assignation (n° of genes), the ubiquity (n° of stations), the environmental parameters associated to their distribution, the preferential diatom size-class owning the clade, the environmental condition enhancing its expression and the geographical areas where it was found more expressed.

| | **Clade** | **Taxa assignation (n° of sequences)** | **DISTRIBUTION** | | | **EXPRESSION** | | |
|---|---|---|---|---|---|---|---|---|
| | | | **Ubiquity** | **Distribution** | **Environmental drivers of distribution** | **Which diatoms** | **Where it is expressed** | **Environmental drivers of modulation in 20-180 µm** |
| **Di*AMT1*** | I | RC (6), PC (78) | 101 | Ubiquitous | / | Big diatoms (20-180 µm) | Across all the basins | $NO_2^-$ (low availability) |
| | II | RC (18), PC (4) | 101 | Ubiquitous | / | Medium small diatoms (0.8-20 µm) | Expressed across all the basins and dominant in MS, NAO and NPO. | Nitrocline depth (lower depths) |
| | III | RC(5), PC(5), RP (36), AP (4) | 104 | Ubiquitous | / | Small-medium diatoms (0.8-20 µm) | Southern oceans (SO, SPO and SAO) and dominant in the IO. | Iron (medium availability) and $NO_2^-$ (low availability) |
| | IV | RC (27), PC(10) | 92 | Ubiquitous | / | Medium-big diatoms (5-180 µm), | Expressed in all the basins except the MS, dominant in SAO and SPO. | / |
| | V | RC (3), PC (21) | 77 | Ubiquitous except MS very oligotrophic stations. | / | / | Relevant abundances in all the basins except MS and IO. | $NO_2^-$ + $NO_3^-$ (medium availabilities) |
| | VI | RC (6), AP (6) | 77 | Ubiquitous except North IO and W-MS | / | / | Specifically expressed in SO and southern IO. | Iron (high abundances) |
| | VII | RC (3) | 8 | Mainly in SO | Temperature (<15°C) | / | Specifically expressed in SO. | / |
| | VIII | RC (5), PC (13), RP (7) | 78 | Absent only in IO and central Atlantic O | $NO_2^-$ + $NO_3^-$ (medium-high availability) and temperature (<15°C) | Small diatoms (0.8-5 µm) | Never particularly abundant, found in the southern regions (SO, SPO, SAO) | / |
| | IX | RC (3), PC (21), RP (13) | 48 | Found on nutrient enriched stations (close to upwelling or SO) | $NO_2^-$ + $NO_3^-$ (medium-high availability) | Small diatoms (0.8-5 µm) | Abundant in the SO, for 0.8-5 µm also in other southern regions (SPO and SAO) | $NO_2^-$ + $NO_3^-$ (high availability) |
| | X | RC (10) | 45 | MS, East-NAO, coastal areas off Argentina and SouthAfrica | Iron (high availability) | / | Specific of the MS. | Iron (high availability) |
| | XI | PC (2) | 7 | Only 4 stations in SAO and NAO. | $NO_2^-$ (high availability) | Small diatoms (0.8-5 µm) | SAO and NAO. | / |

| | | | | | | | | |
|---|---|---|---|---|---|---|---|---|
| ***DiNRT2*** | I | PC (4), RP (36), AP (2) | 92 | Ubiquitous | / | Small-medium diatoms (0.8-20 µm) | Relevant everywhere except SO, dominant in IO, MS and NPO. | $NO_2^-$ + $NO_3^-$ (limited concentrations) |
| | II | RC (9), PC (91), RP (6), AP (1) | 100 | Ubiquitous | / | All size fractions. | Very abundant across all the basins (except SO) | / |
| | III | RP (2) | 25 | NAO and off the S-Africa both in the IO and in the SAO. | $NO_2^-$ + $NO_3^-$ (low concentrations) | Medium-big diatoms (5-180 µm) | Rarely abundant only in the IO | $NO_2^-$ + $NO_3^-$ (medium-high concentrations) and iron (medium-high availability) |
| | IV | PC (5), AP (4) | 50 | Spread across the basins except the Pacific Ocean | Iron (medium-high availability) | Medium diatoms (5-20 µm) | NAO. | Temperature (<17°C) and nitrocline depth (depths >180m) |
| | V | PC (23), RP (3), AP (18) | 96 | Ubiquitous | / | Medium-big diatoms (5-180 µm) | Across all the oceans except SO. | $NO_2^-$ + $NO_3^-$ (low availabilities) |
| | VI | RC (4), PC (16), RP (19), AP (7) | 79 | Ubiquitous except very oligotrophic stations and MS | / | / | Dominant in SO, enriched in several stations of SAO and SPO. | $NO_2^-$ + $NO_3^-$ (medium-high concentrations) |
| | VII | PC (2), RP (1), AP (4) | 44 | Spread in stations across all the basins | / | / | Abundant in very few stations located across all the basins. | / |
| | VIII | RP (2) | 18 | Specific of SPO but also found in three stations spread in NAO, SAO and IO | Iron (low concentrations) | Medium-big diatoms (5-180 µm) | Extremely rare in across NPO,SPO and NAO | / |
| | IX | RC (1), PC (6) | 24 | Few stations spread across the oceans but mainly south Africa and MS. | / | / | Relevant abundances in the MS, in 92 SRF (SPO) and 66 SRF (SAO) | / |
| | X | PC (4), AP (3) | 7 | Only in SO | $NO_2^-$ + $NO_3^-$ (high availability) and temperature (<15°C) | Medium-big diatoms (5-180 µm) | Typical of SO. | / |
| | XI | PC (1), RP (2) | 34 | Only higher latitudes stations, mainly in the Atlantic Ocean | Temperature (15-20°C) | Small diatoms (0.8-5 µm), | Dominant in MS and SPO (only DCM), but present also in NAO, SAO and NPO. | Absent in 20-180 µm |
| | XII | RC (2), PC (2) | 38 | Everywhere except MS and IO | $NO_2^-$ + $NO_3^-$ (medium-high concentration) and $NO_2^-$ (medium availability) | | Relevant abundances only in 4 DCM (NAO) and 100 DCM (SPO) | $NO_2^-$ (high concentrations) |

**Tab. S2. Spearman correlations with environmental variables.** Spearman correlations between the zero-adjusted Bray-Curtis distance between surface and DCM samples at the same station, the sum of Di*NRT2* and Di*AMT1* transcript abundances and the environmental variables available in *Tara* Oceans for the 4 size classes of interest. Only the variable with a significant (adjusted p-value<0.05) correlation that distance in surface or DCM are shown.

| Size class (μm) | N transporter genes variable | Environmental parameter | Environmental parameter depth | Spearman RHO | Adjusted p-value |
|---|---|---|---|---|---|
| **Di*AMT1*** | | | | | |
| **0.8-5** | Zero-adjusted Bray-Curtis distance between SRF and DCM samples | Nitrocline depth | / | -0.6075 | 0.030 |
| **20-180** | Zero-adjusted Bray-Curtis distance between SRF and DCM samples | Monthly ipar | SRF | 0.5119 | 0.027 |
| **20-180** | Zero-adjusted Bray-Curtis distance between SRF and DCM samples | $NH_4^+$ | SRF | 0.5363 | 0.016 |
| **0.8-5** | Zero-adjusted Bray-Curtis distance between SRF and DCM samples | Mean angular scattering coeff (117-470 nm) | DCM | 0.5613 | 0.035 |
| **0.8-5** | mRNA levels | Mean Latitude | SRF | -0.38 | 0.0429 |
| **0.8-5** | mRNA levels | Mean Oxygen | SRF | 0.53 | 0.0018 |
| **0.8-5** | mRNA levels | Mean Temperature | SRF | -0.41 | 0.0219 |
| **0.8-5** | mRNA levels | Oxygen Dissolved | SRF | 0.49 | 0.0033 |
| **0.8-5** | mRNA levels | Silicate | SRF | 0.38 | 0.0443 |
| **0.8-5** | mRNA levels | Temperature | SRF | -0.46 | 0.0081 |
| **20-180** | mRNA levels | Silicate | SRF | 0.37 | 0.029 |
| **180-2000** | mRNA levels | Mean Density | DCM | 0.72 | 0.0305 |
| **20-180** | mRNA levels | Fe (PISCES2) | DCM | 0.64 | 0.0031 |
| **Di*NRT2*** | | | | | |
| **5-20** | Zero-adjusted Bray-Curtis distance between SRF and DCM samples | Mean Flux at 150 m | DCM | -0.6678 | 0.049 |
| **0.8-5** | mRNA levels | Fe (PISCES2) | SRF | 0.39 | 0.0391 |
| **0.8-5** | mRNA levels | Fe (DARWIN) | SRF | 0.52 | 0.0118 |
| **0.8-5** | mRNA levels | fqy2 phi sat | SRF | -0.43 | 0.0189 |
| **0.8-5** | mRNA levels | Lyapunov | SRF | 0.41 | 0.0226 |
| **0.8-5** | mRNA levels | Mean depth max $O_2$ | SRF | 0.51 | 0.0032 |
| **0.8-5** | mRNA levels | Mean Longitude | SRF | 0.45 | 0.0092 |
| **0.8-5** | mRNA levels | Mean Nitrates | SRF | -0.56 | 0.003 |
| **0.8-5** | mRNA levels | Nitrate | SRF | -0.46 | 0.0075 |
| **0.8-5** | mRNA levels | $NO_2^-$ | SRF | -0.49 | 0.0046 |
| **0.8-5** | mRNA levels | $NO_2^-$ (DARWIN) | SRF | -0.4 | 0.0253 |
| **0.8-5** | mRNA levels | $NO_2^-$ $NO_3^-$ | SRF | -0.57 | 0.0004 |
| **0.8-5** | mRNA levels | $NO_3^-$ (DARWIN) | SRF | -0.58 | 0.0002 |
| **0.8-5** | mRNA levels | Oxygen Saturation | SRF | 0.39 | 0.0326 |
| **0.8-5** | mRNA levels | Oxygen Utilization | SRF | -0.43 | 0.015 |
| **0.8-5** | mRNA levels | $PO_4^{3-}$ | SRF | -0.43 | 0.0158 |
| **0.8-5** | mRNA levels | Ratio $NO_3^-/NH_4^+$ (DARWIN) | SRF | -0.63 | 0.00002 |
| **0.8-5** | mRNA levels | Temperature Seasonality index | SRF | 0.37 | 0.0467 |
| **20-180** | mRNA levels | Fe (PISCES2) | SRF | 0.36 | 0.0382 |
| **20-180** | mRNA levels | Mean Nitrates | SRF | -0.48 | 0.0059 |
| **20-180** | mRNA levels | $NH_4^+$ (DARWIN) | SRF | -0.34 | 0.0485 |
| **20-180** | mRNA levels | Nitrate | SRF | -0.36 | 0.0378 |
| **20-180** | mRNA levels | $NO_2^-$ | SRF | -0.4 | 0.0166 |
| **20-180** | mRNA levels | $NO_2^-$ $NO_3^-$ | SRF | -0.53 | 0.0003 |
| **20-180** | mRNA levels | $PO_4^{3-}$ | SRF | -0.45 | 0.0051 |
| **5-20** | mRNA levels | $NO_2^-$ $NO_3^-$ | SRF | -0.46 | 0.0072 |
| **5-20** | mRNA levels | $PO_4^{3-}$ | SRF | -0.37 | 0.0489 |
| **0.8-5** | mRNA levels | Mean Chloro | DCM | -0.67 | 0.0013 |
| **0.8-5** | mRNA levels | Nitrate | DCM | -0.5 | 0.0413 |
| **0.8-5** | mRNA levels | Phosphate (100 m) | DCM | -0.61 | 0.0211 |
| **0.8-5** | mRNA levels | Nitrates Seasonality index | DCM | -0.57 | 0.01366 |
| **20-180** | mRNA levels | Mean Chloro | DCM | -0.59 | 0.0083 |
| **20-180** | mRNA levels | Mean Density | DCM | 0.5 | 0.0448 |
| **20-180** | mRNA levels | Mean Depth Max $O_2$ | DCM | 0.61 | 0.0077 |
| **20-180** | mRNA levels | Mean Nitrates | DCM | -0.59 | 0.01918 |
| **20-180** | mRNA levels | $NO_2^-$ | DCM | -0.58 | 0.01099 |
| **20-180** | mRNA levels | $NO_2^-$ $NO_3^-$ | DCM | -0.6 | 0.0075 |
| **20-180** | mRNA levels | Phosphate | DCM | -0.49 | 0.0441 |

| | | | | | |
|---|---|---|---|---|---|
| **20-180** | mRNA levels | Phosphate (100 m) | DCM | -0.58 | 0.0253 |
| **20-180** | mRNA levels | $PO_4^{3-}$ | DCM | -0.62 | 0.0045 |
| **20-180** | mRNA levels | SI | DCM | -0.5 | 0.0389 |

**Tab. S3. Enzyme names and definitions for the KEGG orthologous groups (KO).** All the KO investigated resulting having a significant correlation with at least one diatom N transporter clade are herein defined.

| KEGG orthologous group (KO) | Definition: |
|---|---|
| K01455 | formamidase; |
| K02575 | MFS transporter, NNP family, nitrate/nitrite transporter; |
| K15576 | nitrate/nitrite transport system substrate-binding protein; |
| K15577 | nitrate/nitrite transport system permease protein; |
| K15578 | nitrate/nitrite transport system ATP-binding protein; |
| K15579 | nitrate/nitrite transport system ATP-binding protein; |
| K00367 | ferredoxin-nitrate reductase; |
| K10534 | nitrate reductase (NAD(P)H); |
| K00370 | nitrate reductase / nitrite oxidoreductase, alpha subunit; |
| K00371 | nitrate reductase / nitrite oxidoreductase, beta subunit; |
| K00374 | nitrate reductase gamma subunit; |
| K00372 | assimilatory nitrate reductase catalytic subunit; |
| K00360 | assimilatory nitrate reductase electron transfer subunit; |
| K02567 | periplasmic nitrate reductase NapA; |
| K02568 | cytochrome c-type protein NapB; |
| K17877 | nitrite reductase (NAD(P)H); |
| K00362 | nitrite reductase (NADH) large subunit; |
| K00363 | nitrite reductase (NADH) small subunit; |
| K00366 | ferredoxin-nitrite reductase; |
| K03385 | nitrite reductase (cytochrome c-552); |
| K15876 | cytochrome c nitrite reductase small subunit; |
| K00368 | nitrite reductase (NO-forming); |
| K15864 | nitrite reductase (NO-forming) / hydroxylamine reductase; |
| K04561 | nitric oxide reductase subunit B; |
| K02305 | nitric oxide reductase subunit C; |
| K15877 | fungal nitric oxide reductase; |
| K00376 | nitrous-oxide reductase; |
| K02586 | nitrogenase molybdenum-iron protein alpha chain; |
| K02591 | nitrogenase molybdenum-iron protein beta chain; |
| K02588 | nitrogenase iron protein NifH; |
| K00531 | nitrogenase delta subunit; |
| K20932 | hydrazine synthase subunit; |
| K20933 | hydrazine synthase subunit; |
| K20934 | hydrazine synthase subunit; |
| K20935 | hydrazine dehydrogenase; |
| K10944 | methane/ammonia monooxygenase subunit A; |
| K10945 | methane/ammonia monooxygenase subunit B; |

| | |
|---|---|
| K10946 | methane/ammonia monooxygenase subunit C; |
| K05601 | hydroxylamine reductase; |
| K10535 | hydroxylamine dehydrogenase; |
| K00459 | nitronate monooxygenase; |
| K19823 | nitroalkane oxidase; |
| K01501 | nitrilase; |
| K15371 | glutamate dehydrogenase; |
| K00260 | glutamate dehydrogenase; |
| K00261 | glutamate dehydrogenase (NAD(P)+); |
| K00262 | glutamate dehydrogenase (NADP+); |
| K01915 | glutamine synthetase; |
| K00264 | glutamate synthase (NADPH/NADH); |
| K00265 | glutamate synthase (NADPH/NADH) large chain; |
| K00266 | glutamate synthase (NADPH/NADH) small chain; |
| K00284 | glutamate synthase (ferredoxin); |
| K01948 | carbamoyl-phosphate synthase (ammonia); |
| K01725 | cyanate lyase; |
| K00926 | carbamate kinase; |
| K01672 | carbonic anhydrase; |
| K18245 | carbonic anhydrase 2; |
| K18246 | carbonic anhydrase 4; |
| K01673 | carbonic anhydrase; |
| K01674 | carbonic anhydrase |
