# Supplementary figures and images for: Meta-Omics Reveals Genetic Flexibility of Diatom Nitrogen Transporters in Response to Environmental Changes
Source: Mol Biol Evol. 2019 Jul 1;36(11):2522–35. doi: 10.1093/molbev/msz157 (PMC6805229; doi:10.1093/molbev/msz157)

**A**

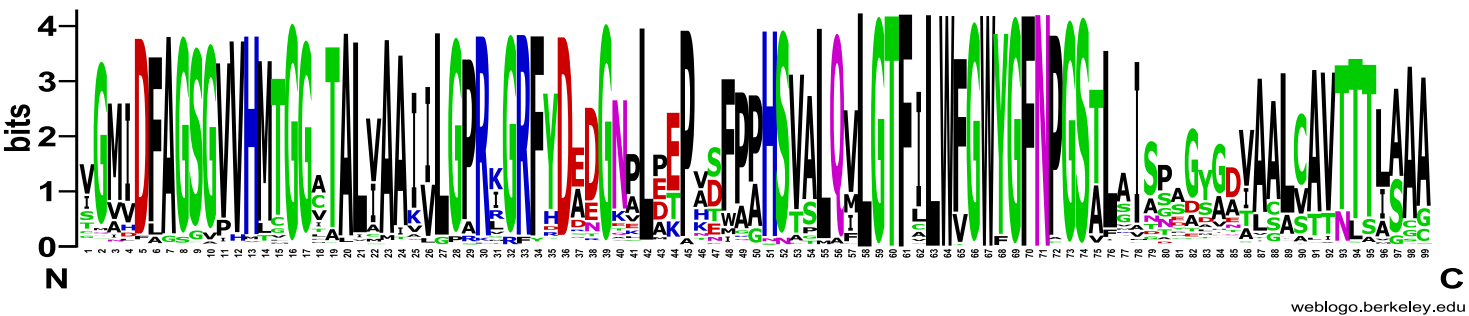

# B

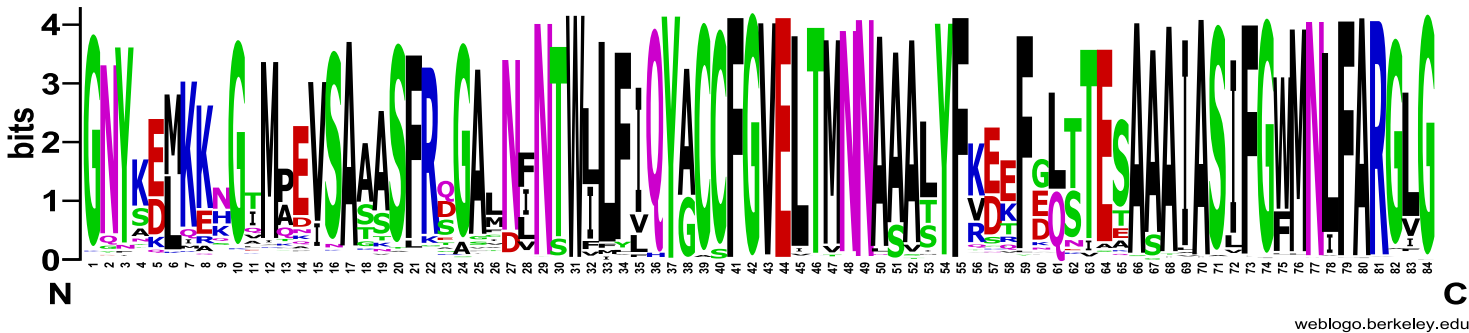

C

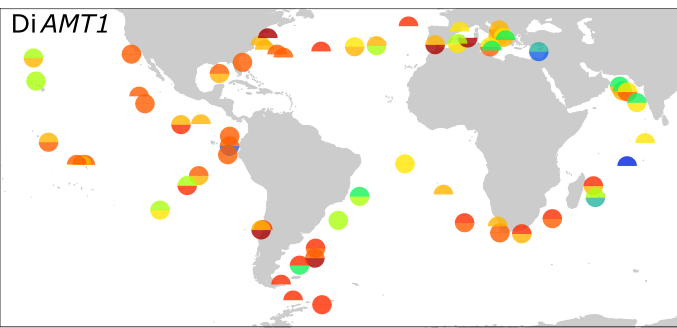

D

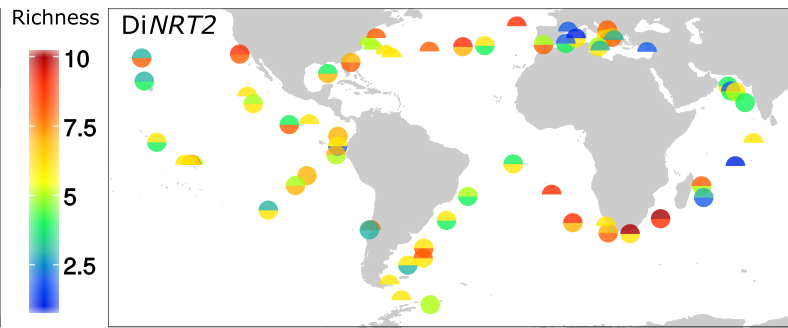

Supplement: msz157_Supplementary_Data [file msz157_supplementary_data.zip › Fig_S1_conserved_regions_richness.pdf]

**A****DiAMT1**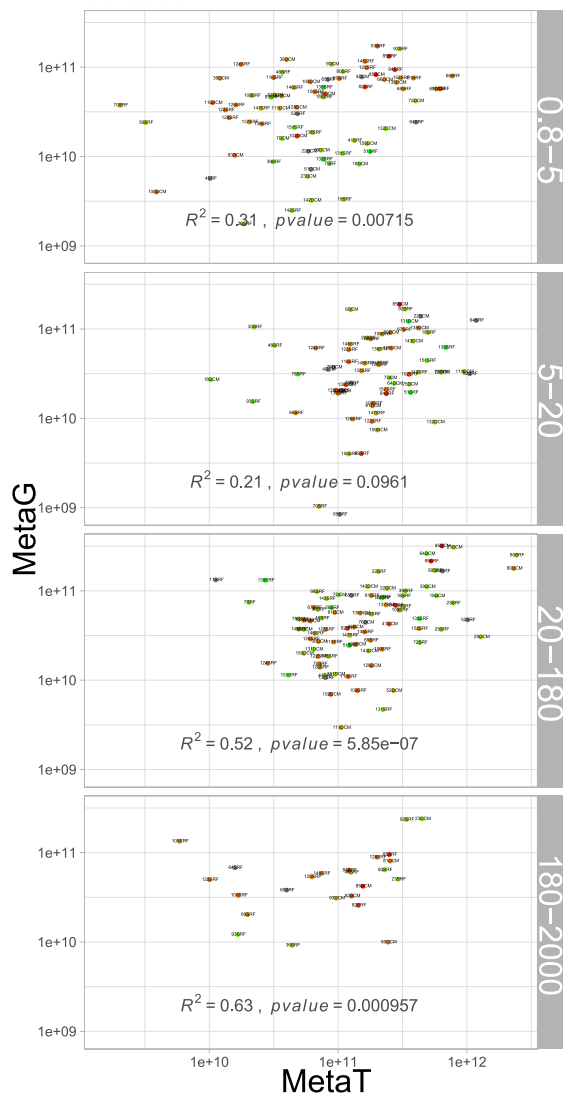**DiNRT2**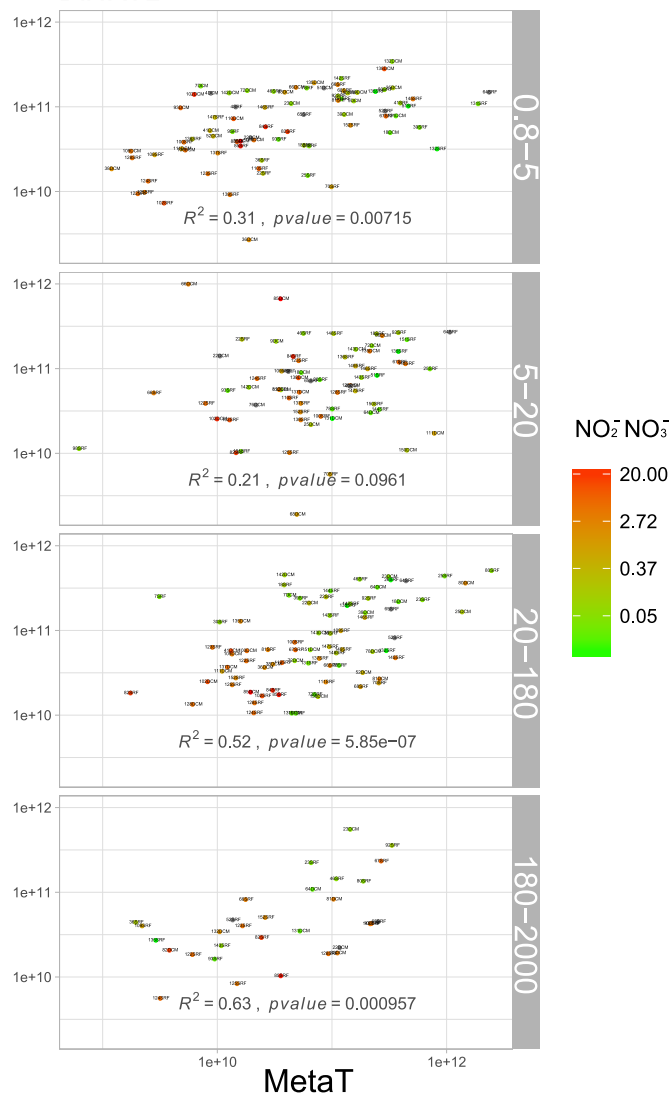**B****DiAMT1**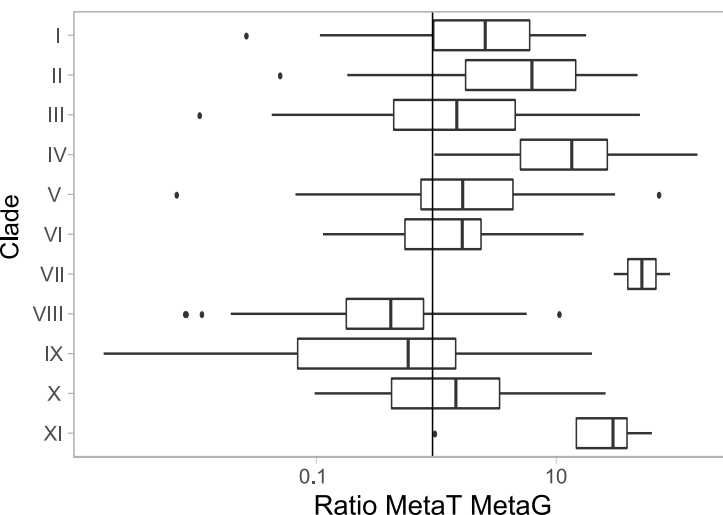**DiNRT2**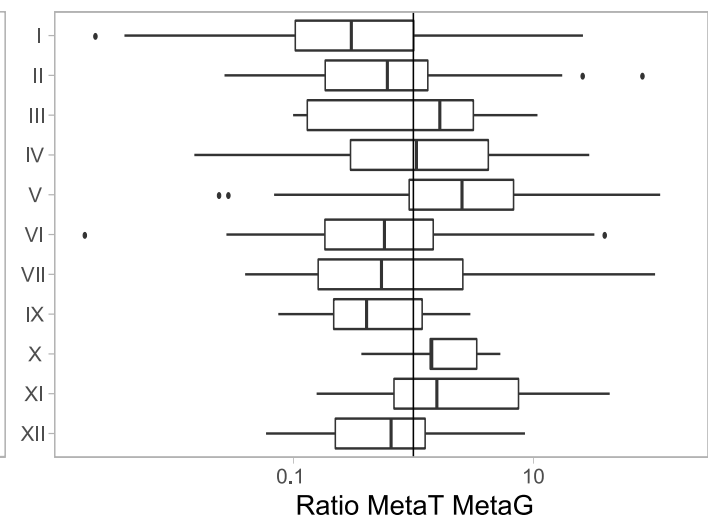

Supplement: msz157_Supplementary_Data [file msz157_supplementary_data.zip › Fig_S2_gene_mrna_ratio.pdf]

**A**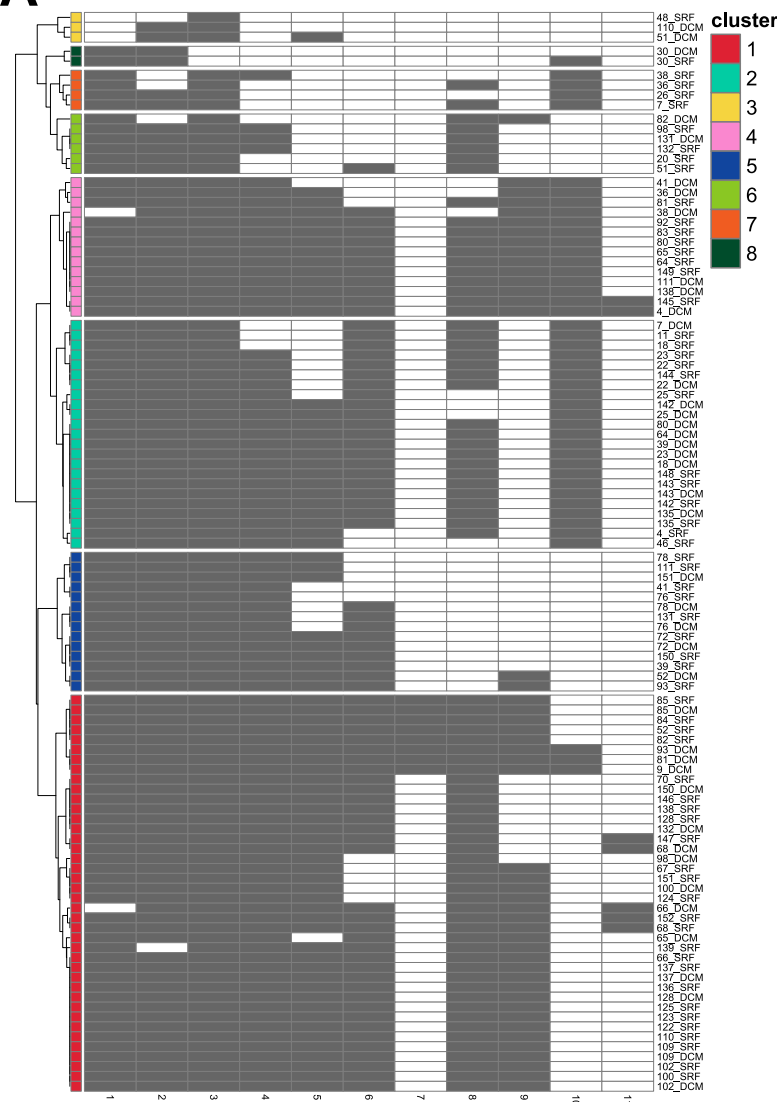**B**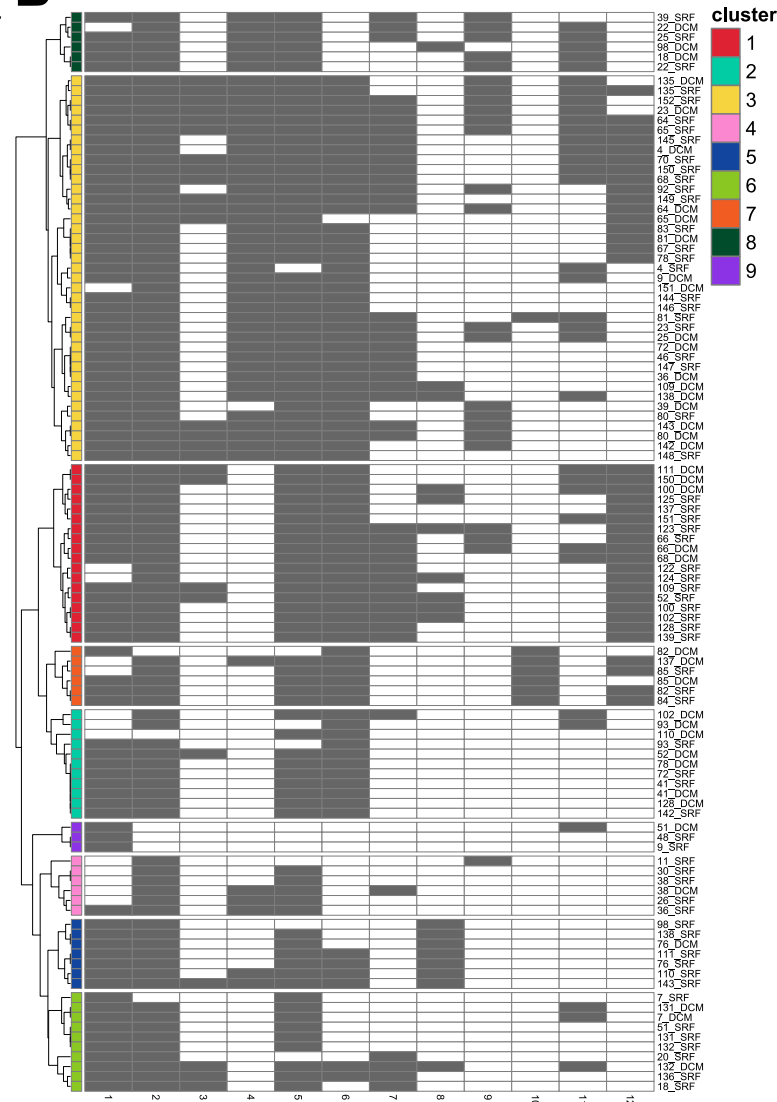**C**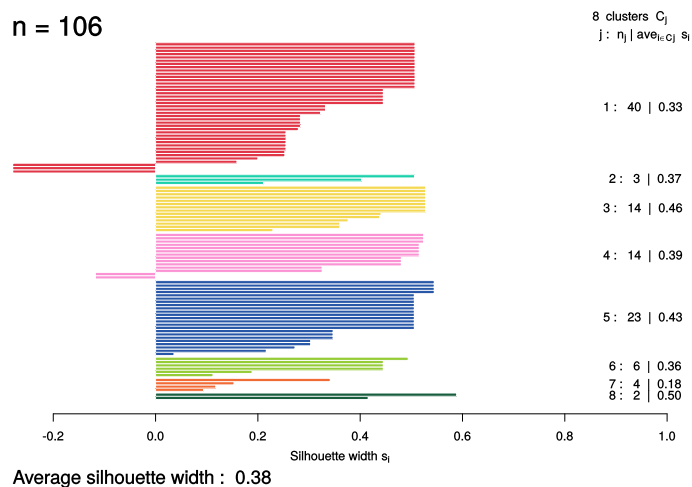**D**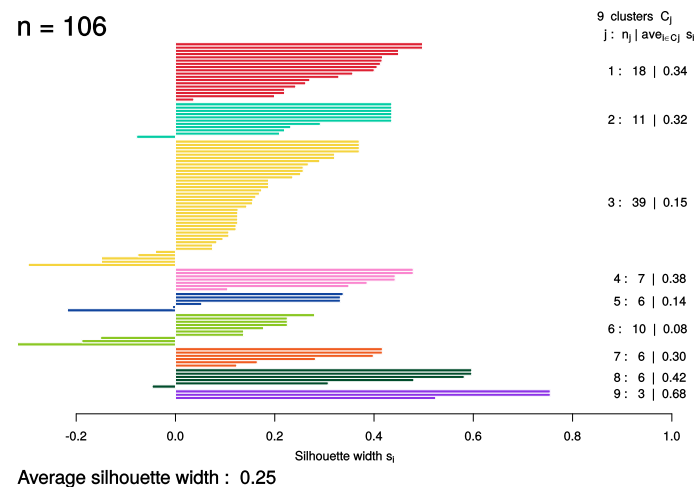

Supplement: msz157_Supplementary_Data [file msz157_supplementary_data.zip › Fig_S3_clades_presence_absence.pdf]

**A**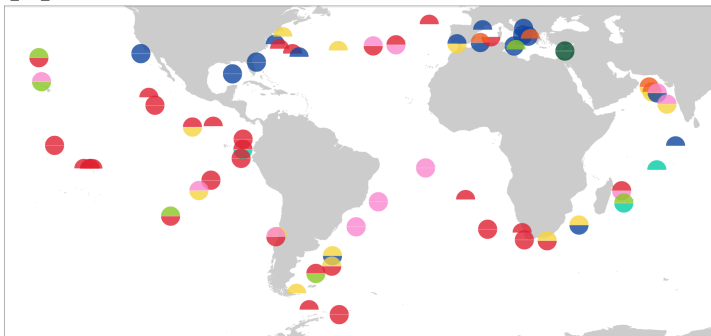**B**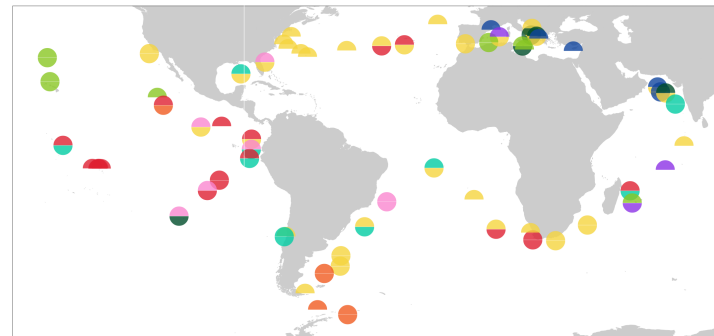**C**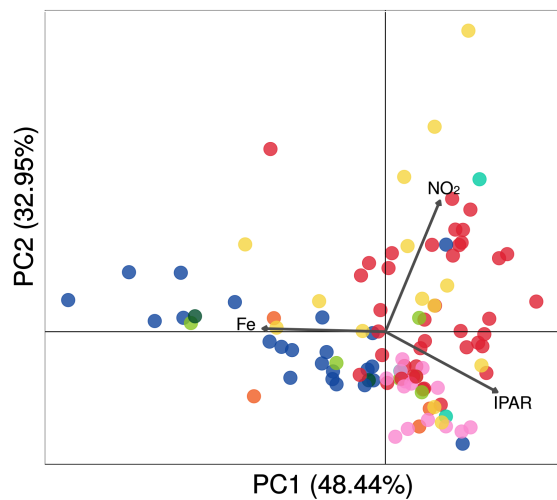**D**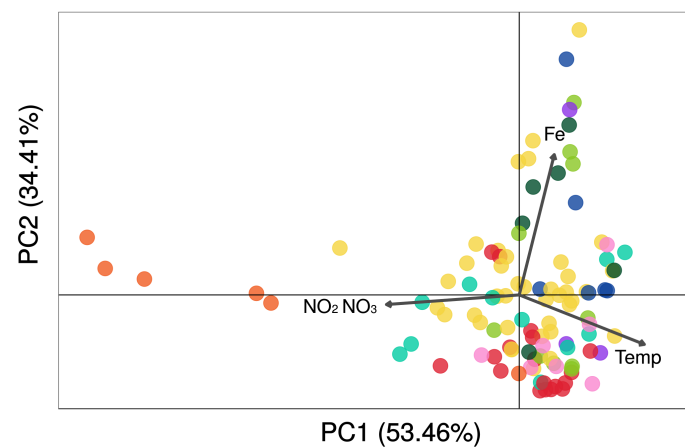**E**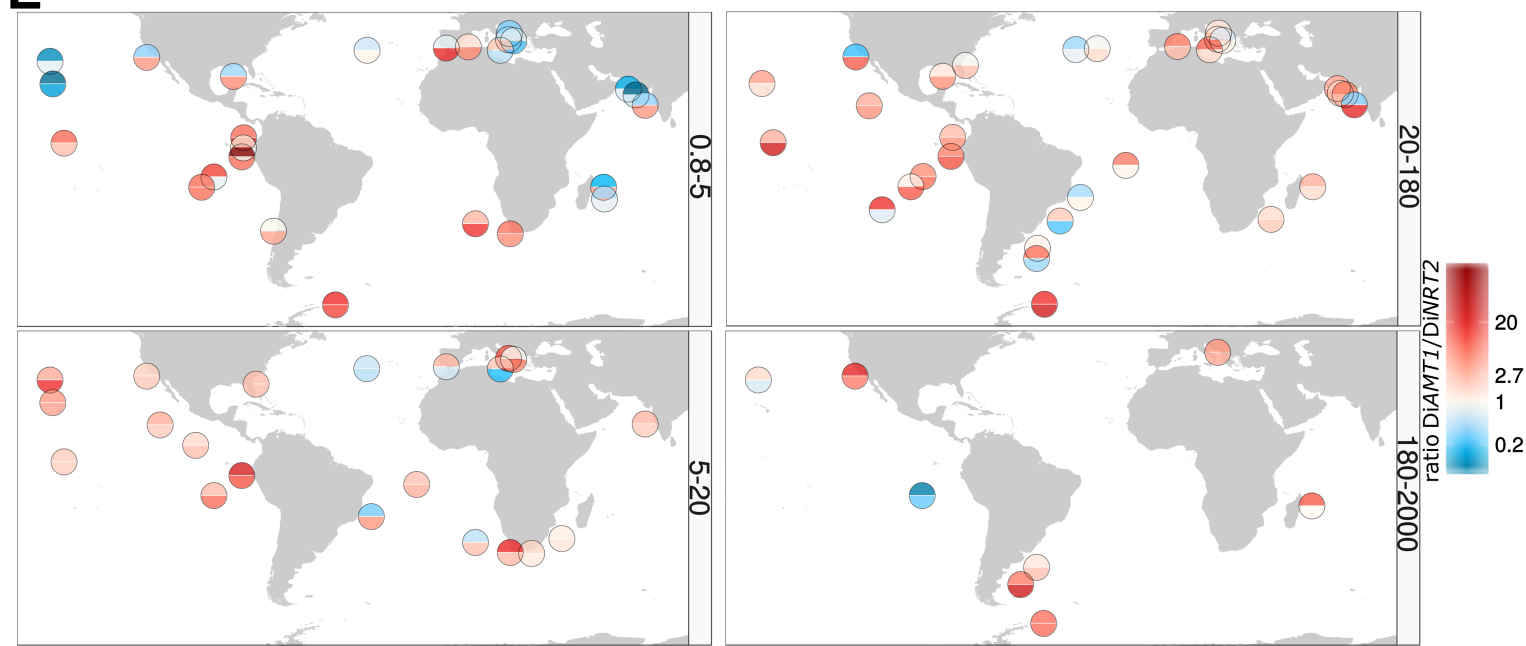

Supplement: msz157_Supplementary_Data [file msz157_supplementary_data.zip › Fig_S4_clades_biogeographies.pdf]

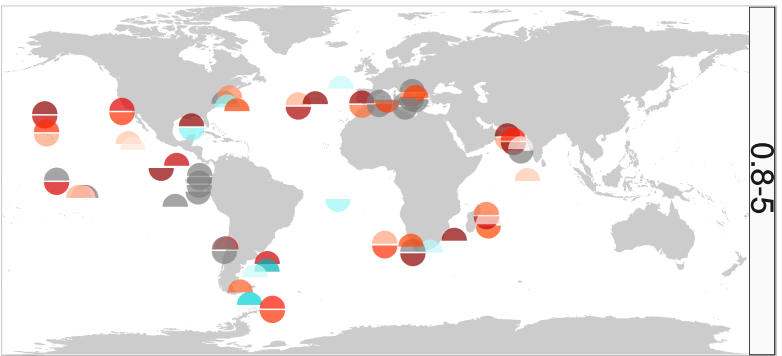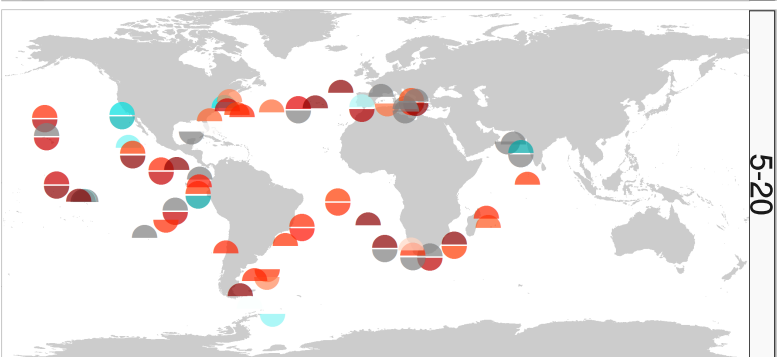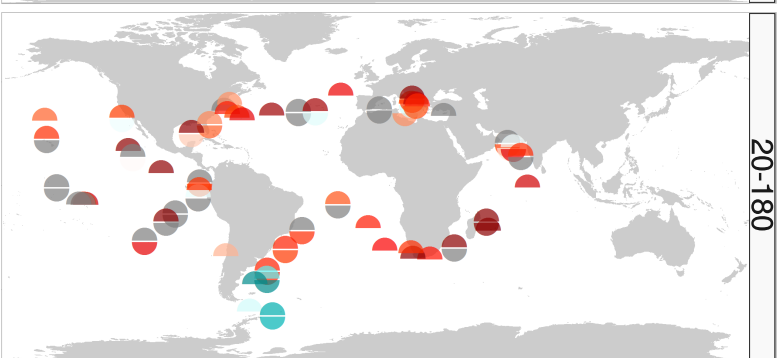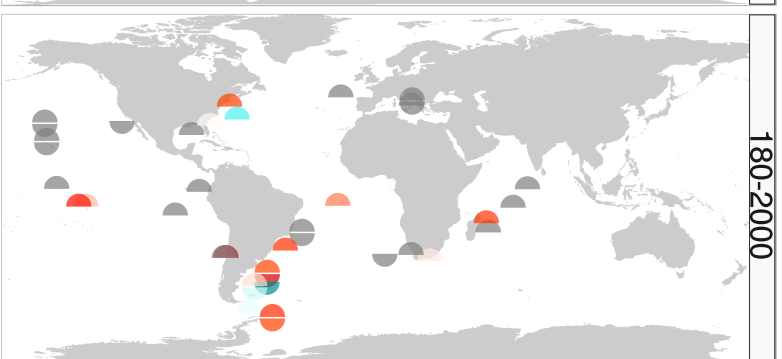

VM/PM

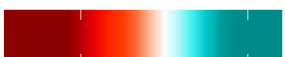

50.0

1

0.2

Supplement: msz157_Supplementary_Data [file msz157_supplementary_data.zip › Fig_S5_vm_pm_ratio.pdf]

AMT1

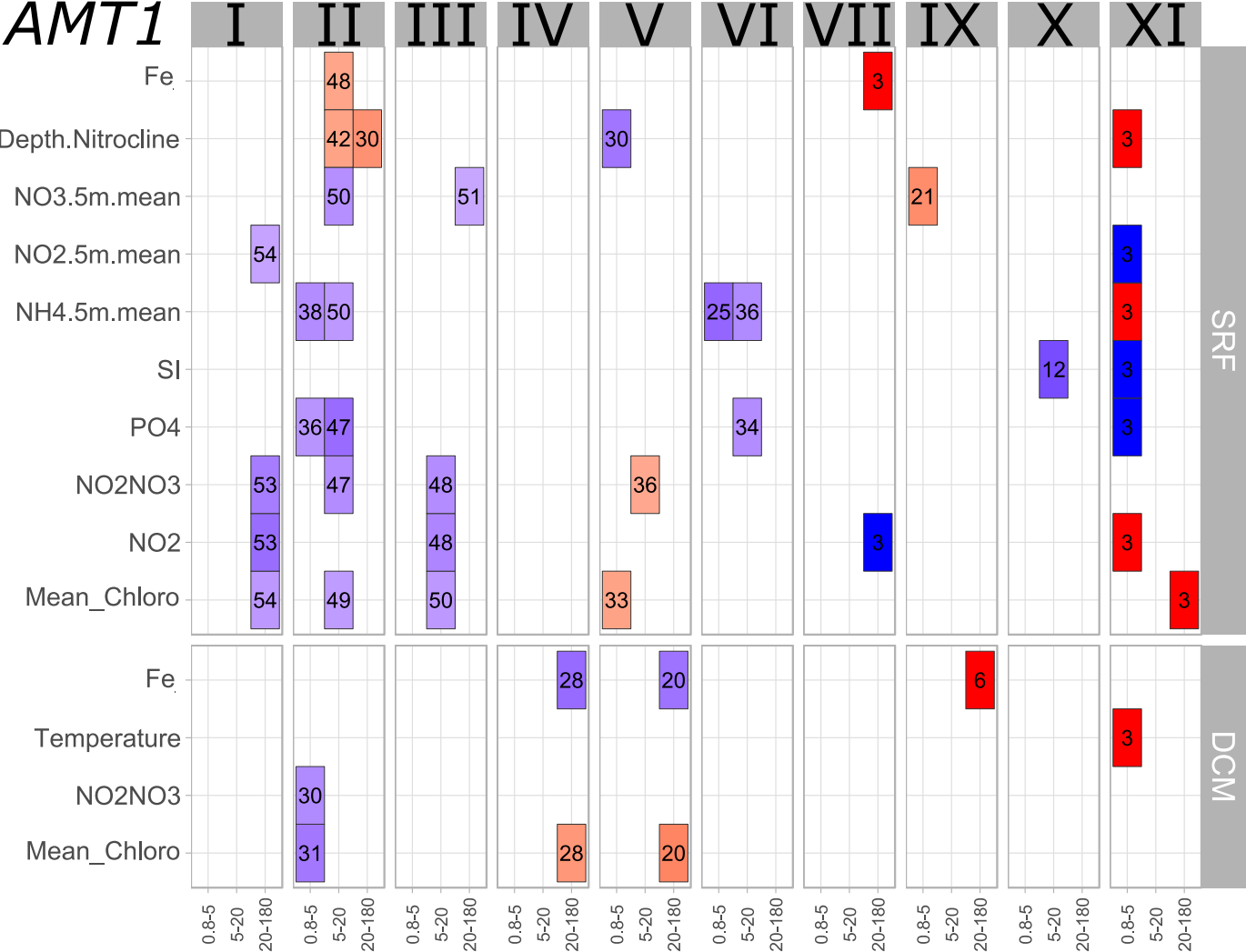

NRT2

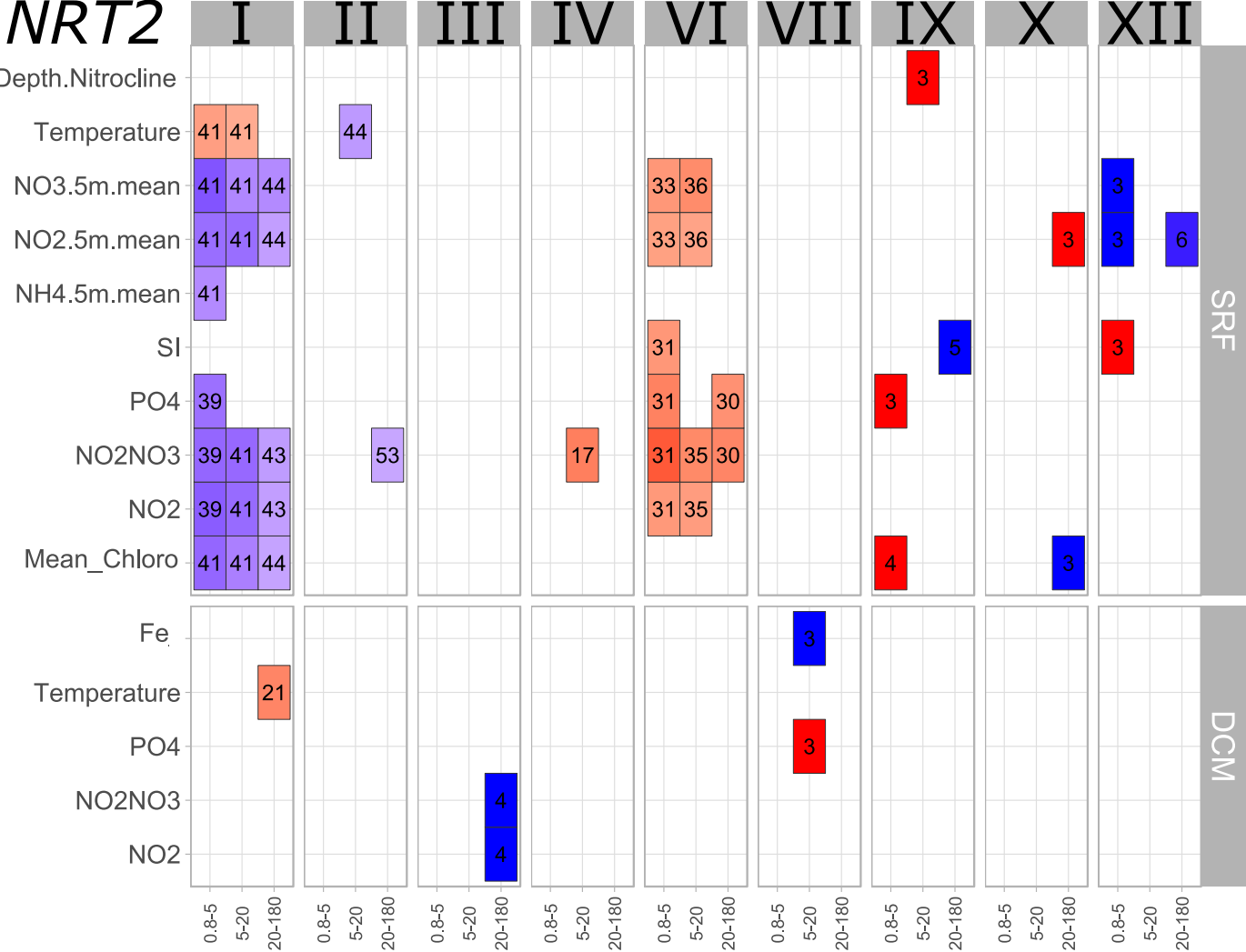

Supplement: msz157_Supplementary_Data [file msz157_supplementary_data.zip › Fig_S6_environmental_correlations.pdf]

**A**

Relative contribution

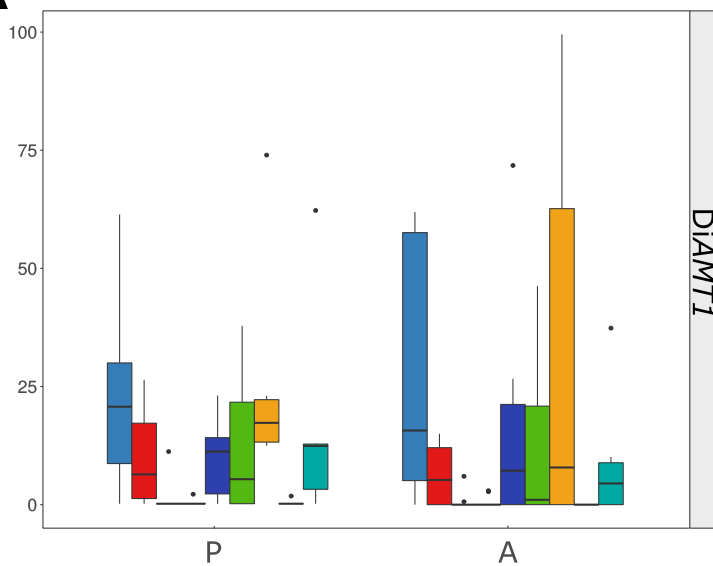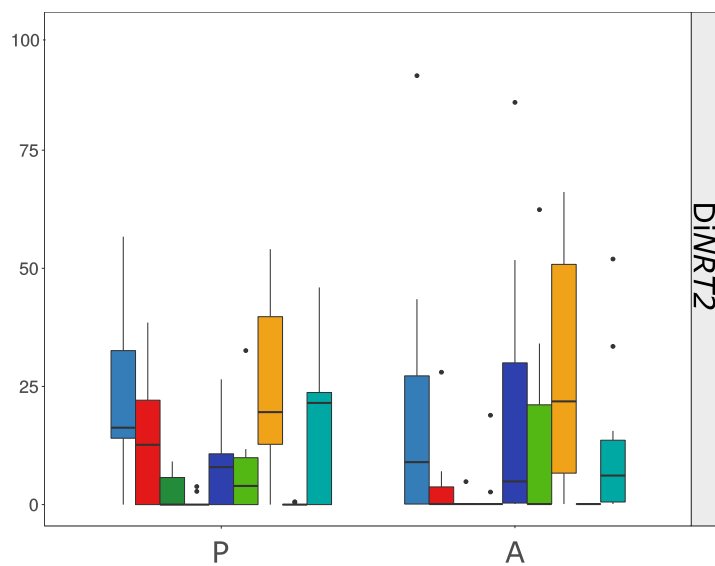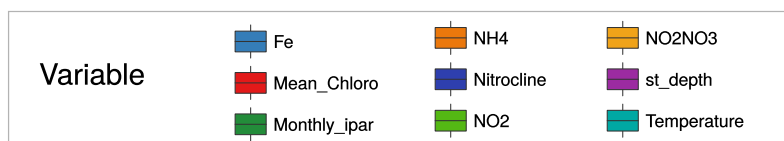**B**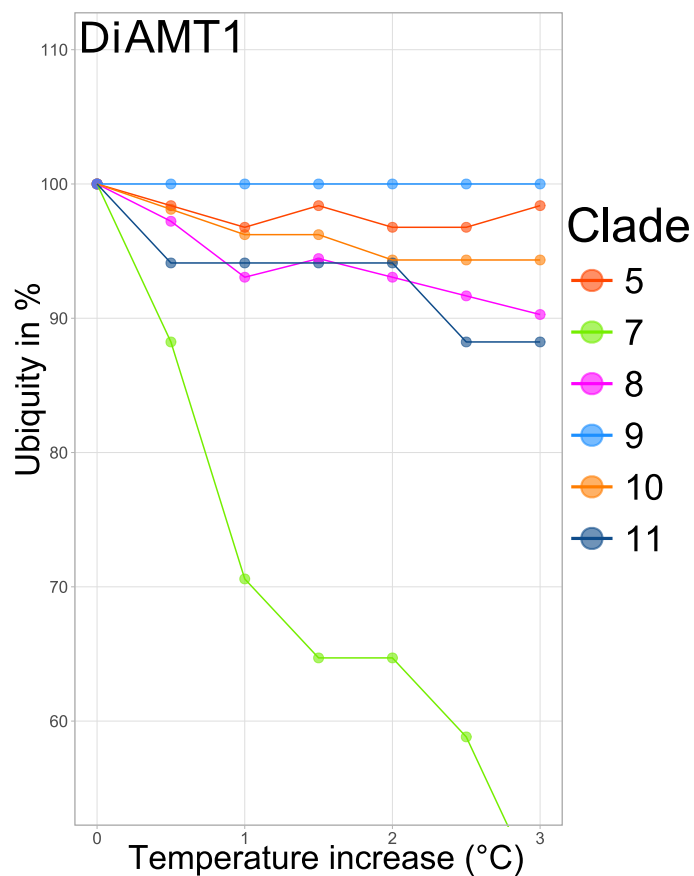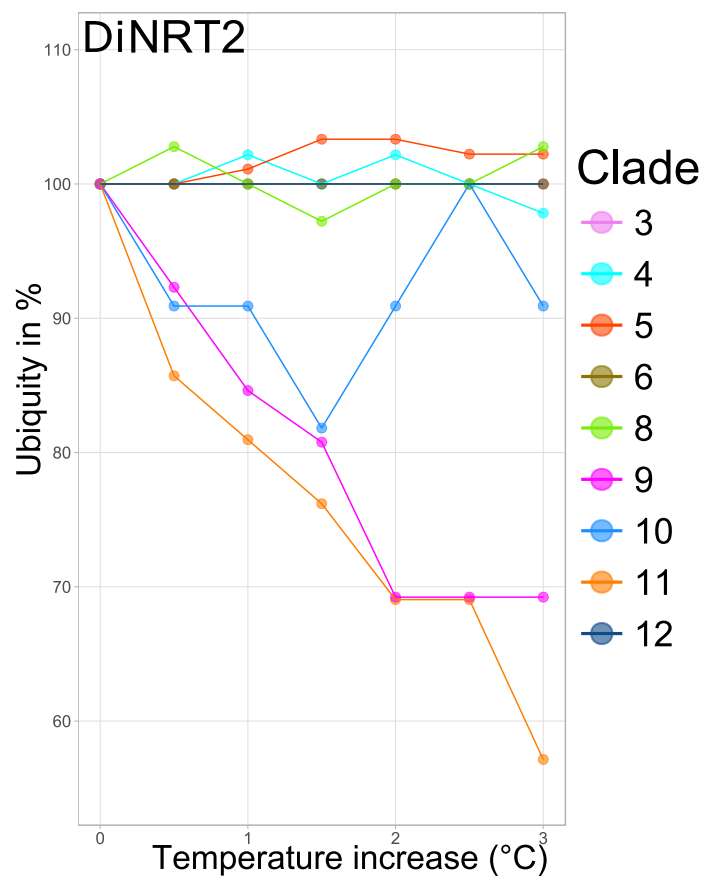

Supplement: msz157_Supplementary_Data [file msz157_supplementary_data.zip › Fig_S7_brt_results.pdf]

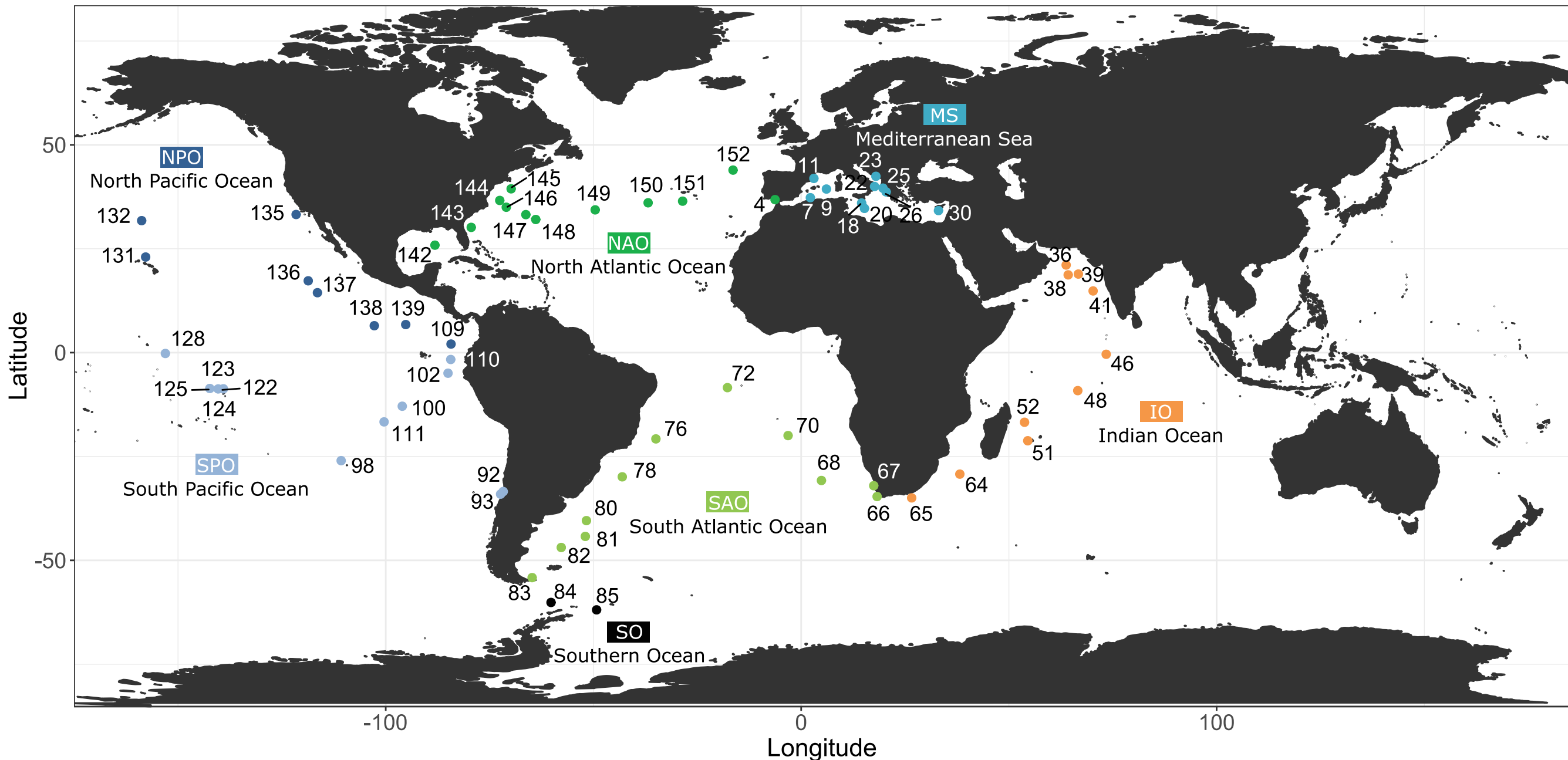

Supplement: msz157_Supplementary_Data [file msz157_supplementary_data.zip › Fig_S8_tara_stations.pdf]
